# Efficacy of live oral rotavirus vaccines by duration of follow-up: a meta-regression of randomised controlled trials

**DOI:** 10.1016/S1473-3099(19)30126-4

**Published:** 2019-07

**Authors:** Andrew Clark, Kevin van Zandvoort, Stefan Flasche, Colin Sanderson, Julie Bines, Jacqueline Tate, Umesh Parashar, Mark Jit

**Affiliations:** a London School of Hygiene and Tropical Medicine, London, UK; b Murdoch Children's Research Institute, Melbourne, VIC, Australia; c Department of Paediatrics, The University of Melbourne, Melbourne, VIC, Australia; d Department of Gastroenterology and Clinical Nutrition, Royal Children's Hospital, Melbourne, VIC, Australia; e Centers for Disease Control and Prevention, Atlanta, GA, USA; f Modelling and Economics Unit, Public Health England, London, UK

## Abstract

**Background:**

The duration of protection offered by rotavirus vaccines varies across the world, and this variation is important to understanding and predicting the effects of the vaccines. There is now a large body of evidence on the efficacy of live oral rotavirus vaccines in different settings, but these data have never been synthesised to obtain robust estimates of efficacy by duration of follow-up. Our aim is to estimate the efficacy of live oral rotavirus vaccines at each point during follow-up and by mortality stratum.

**Methods:**

In our meta-regression study, we identified all randomised controlled trials of rotavirus vaccines published until April 4, 2018, using the results of a Cochrane systematic review, and cross checked these studies against those identified by another systematic review. We excluded trials that were based on special populations, trials without an infant schedule, and trials without clear reporting of numbers of enrolled infants and events in different periods of follow-up. For all reported periods of follow-up, we extracted the mean duration of follow-up (time since administration of the final dose of rotavirus vaccination), the number of enrolled infants, and case counts for rotavirus-positive severe gastroenteritis in both non-vaccinated and vaccinated groups. We used a Bayesian hierarchical Poisson meta-regression model to estimate the pooled cumulative vaccine efficacy (VE) and its waning with time for three mortality strata. We then converted these VE estimates into instantaneous VE (iVE).

**Findings:**

In settings with low mortality (15 observations), iVE pooled for infant schedules of Rotarix and RotaTeq was 98% (95% credibility interval 93–100) 2 weeks following the final dose of vaccination and 94% (87–98) after 12 months. In medium-mortality settings (11 observations), equivalent estimates were 82% (74–92) after 2 weeks and 77% (67–84) after 12 months. In settings with high mortality (24 observations), there were five different vaccines with observation points for infant schedules. The pooled iVE was 66% (48–81) after 2 weeks of follow-up and 44% (27–59) after 12 months.

**Interpretation:**

Rotavirus vaccine efficacy is lower and wanes more rapidly in high-mortality settings than in low-mortality settings, but the earlier peak age of disease in high-mortality settings means that live oral rotavirus vaccines are still likely to provide substantial benefit.

**Funding:**

Bill & Melinda Gates Foundation.

## Introduction

Rotavirus gastroenteritis is estimated to cause around 200 000 child deaths each year,^1^, ^2^, ^3^ mostly in sub-Saharan Africa and south Asia. Episodes of rotavirus gastroenteritis occur frequently in young children irrespective of living standards and are a major contributor to health-care costs worldwide.^4^, ^5^

More than half of the countries in the world have introduced rotavirus vaccines into their national immunisation programmes.^6^ Infants typically receive two oral doses of Rotarix (GlaxoSmithKline Biologicals, London, UK) or three oral doses of RotaTeq (Merck & Co, Kenilworth, NJ, USA) in the first 6 months of life.^7^, ^8^ Both vaccines have shown high and durable efficacy against episodes of severe rotavirus gastroenteritis in high-income settings but lower and less durable efficacy in sub-Saharan Africa and south Asia.^9^, ^10^, ^11^, ^12^ Other live oral rotavirus vaccines are becoming available, such as ROTAVAC (Bharat Biotech, Hyderabad, India), ROTASIIL (Serum Institute of India, Pune, India), and RV3-BB (Murdoch Children's Research Institute, Melbourne, Australia), but these vaccines have also reported low or waning efficacy in high-mortality settings (eg, India, Indonesia, and Niger) when used as part of a standard infant schedule.^13^, ^14^, ^15^, ^16^ Alternative schedules are being considered as one way to improve efficacy in the second year of life. Alternatives might involve administering the first dose at birth^15^ or administering a booster dose at age 9–12 months.^17^

Countries considering the introduction of rotavirus vaccine, global bodies such as WHO, and donors funding vaccine introduction in resource-poor settings require accurate projections of the potential effect of vaccination. Such projections are also useful in surveillance after vaccine introduction, to ensure that the vaccine is performing as expected and to estimate the remaining burden of disease after the vaccine has been introduced. Mathematical models can predict the potential effect of rotavirus vaccines but require credible estimates of vaccine efficacy by duration of follow-up in different settings. This information is also crucial to the evaluation of alternative vaccination schedules. A large body of evidence now exists from high-quality randomised controlled trials (RCTs) in different parts of the world, but these data have never been pooled and synthesised to obtain robust estimates of vaccine efficacy by duration of follow-up. Combining this evidence is not straightforward. There is substantial variation in trial settings, follow-up periods, sample sizes, case definitions, and statistical methods used to calculate CIs. In addition, the main outcome reported in RCTs is the cumulative vaccine efficacy (VE) over a period of many weeks, but if there is evidence of vaccine waning, then the cumulative efficacy over the entire follow-up period might be different to the actual instantaneous VE (iVE) at different times within that period of follow-up.

**Research in context**

**Evidence before the study**

In a Cochrane systematic review published in 2012, Soares-Weiser and colleagues identified 11 randomised controlled trials that showed that live oral rotavirus vaccines induce high and durable efficacy against episodes of severe rotavirus gastroenteritis in high-income settings, but lower and less durable efficacy in sub-Saharan Africa and south Asia.

**Added value of this study**

To our knowledge, this is the first study in which all the available evidence from randomised controlled trials has been synthesised to obtain robust estimates of efficacy by duration of follow-up. We include several new data points from Asia and use a novel approach to convert cumulative vaccine efficacy into instantaneous vaccine efficacy. Our analysis provides the most comprehensive evidence to date that rotavirus vaccine efficacy is lower and wanes more rapidly in high-mortality settings than in low-mortality settings. We show that, in Indonesia, a neonatal schedule provides more durable protection than the standard infant schedule, although this analysis was based on very few case counts in each week of follow-up.

**Implications of all the available evidence**

Live oral rotavirus vaccines are likely to provide substantial benefit globally. In high-mortality settings, strategies to optimise the effect of rotavirus vaccination warrant serious consideration. Estimates of the instantaneous efficacy of live oral rotavirus vaccines by duration of follow-up will be crucial to understanding the potential effect of alternative rotavirus vaccination schedules in different countries.

Our aim is to estimate the instantaneous efficacy of live oral rotavirus vaccines by duration of follow-up (time since administration of the final dose of rotavirus vaccination) and by mortality strata.

## Methods

### Search strategy and selection criteria

We ran a meta-regression, in which we included all individual RCTs that were identified in a Cochrane systematic review of studies published until April 8, 2018,^18^ and cross-checked the list against the studies identified by a review by Lamberti and colleagues.^19^ AC obtained the list of RCTs from the authors of the Cochrane review, cross-checked the list against the studies identified by a review by Lamberti and colleagues, extracted relevant data, and contacted the lead investigators of the study where further clarification was needed. A full assessment of the risks of bias associated with each rotavirus vaccine efficacy trial is described in detail in the Cochrane review.^18^ We excluded trials that were based on special populations, trials without an infant schedule, and trials without clear reporting of enrolled infants and events in different periods of follow-up. The outcome measure was efficacy against episodes of severe rotavirus gastroenteritis, which is the primary endpoint reported in nearly all RCTs of rotavirus vaccines. Severe rotavirus gastroenteritis is defined as 11–20 points on the Vesikari scale,^20^ or for some older trials, 15–24 points on the Clark scale.^21^ If this outcome was not reported, we used the closest available proxy, such as efficacy against episodes of rotavirus gastroenteritis, which involved admission to hospital or the emergency department. In all studies, rotavirus-positive episodes were detected by enzyme immunoassay.

This study was approved by the ethics committee (Ref 15829) of the London School of Hygiene and Tropical Medicine.

### Follow-up definition

We extracted vaccine efficacy for all reported periods of follow-up. Most of the studies reported results at two follow-up points. However, we also included studies with a single follow-up point. We extracted the number of individuals eligible for per-protocol analysis and the number of rotavirus-positive cases in both the non-vaccinated and vaccinated groups. We also extracted the mean duration of follow-up in months. We extracted per-protocol estimates because they exclude any disease cases reported in the first 14 days after vaccination and include only infants who received all recommended doses. We added 14 days to the reported mean duration of follow-up to calculate the entire period between administration of the last dose and the mean age at follow-up. If all infants in a study were followed up to a specific age (eg, 12 months), we subtracted the mean age of administration of the final dose (or target age if the mean was not reported) from the specific follow-up age.

### Stratification of studies

To account for heterogeneity between the RCT sites, we grouped all 201 countries in the world into quintiles (very low mortality, low mortality, medium mortality, high mortality, and very high mortality) using the under-5 mortality rate reported for the period 2010–15 in the 2017 revision of the UN Population Division database.^22^ We further collapsed the very low and low quintiles and the high and very high quintiles to give three strata for deaths under age 5 years per 1000 livebirths: low (<13·5 deaths per 1000 livebirths), medium (13·5–28·1 deaths per 1000 livebirths), and high (>28·1 deaths per 1000 livebirths). Each RCT was then assigned to a specific stratum. For RCTs with multiple sites across several countries, we included each individual country as a separate observation point when this was possible. If RCT results were not disaggregated by country, we used the sample size in each site to calculate a weighted under-5 mortality rate and used this estimate to assign the trial to a specific mortality stratum. We restricted the pooled analysis to infant schedules only.

### Recalculating cumulative vaccine efficacy for reported periods of follow-up

We observed substantial variation in the way authors estimated VE and 95% confidence intervals. Our pooled analyses used case counts and sample sizes reported in trials to generate credible intervals, but to ensure consistent reporting of the data in the summary table and plots, we also recalculated VE and 95% confidence intervals using the method of Daly and Altman.^23^, ^24^ VE was calculated as 1 – relative risk (RR) with zero inflation to 0·5.^25^

### Efficacy by duration of follow-up and mortality strata

We used a Bayesian hierarchical meta-regression model to estimate cumulative VE by duration of follow-up. We generated separate pooled estimates for RCTs in low mortality, medium mortality, and high mortality strata. We assumed that errors around the observed numbers of cases in the unvaccinated and vaccinated groups followed Poisson distributions. The total number of cases in the unvaccinated group in study *i* and period *p*, termed Y_i,p,u_, was estimated using the following generalised linear model:

$$\log\left({\text{Y}}_{\text{i},\text{p},\text{u}}\right)=\lambda_{\text{i},\text{p}}+\log\left({\text{P}}_{\text{i},\text{p},\text{u}}\right)$$

Similarly, the total number of cases in the vaccinated group in study *i* and period *p*, termed Y_i,p,v_, was estimated using:

$$\log\left({\text{Y}}_{\text{i},\text{p},\text{v}}\right)=\lambda_{\text{i},\text{p}}+\log\left({\text{P}}_{\text{i},\text{p},\text{v}}\right)+\theta_{\text{i}}\left({\text{t}}_{\text{i},\text{p}}\right)$$

in which λ_i,p_ is the baseline rate of becoming infected, P_i,p,v_ and P_i,p,u_ are the total person-months of follow-up in the vaccinated and unvaccinated group, respectively, and θ_i_(t_i,p_) is the cumulative relative risk (RR) in study i, at t months of follow-up. We used the log of the person-time to estimate the log of the number of cases, so that the person-time was used on its identity scale when converting the cases to the identity scale. Total person-months of follow-up was calculated as the number of participants at the beginning of the follow-up period multiplied by the reported mean duration of follow-up. The hierarchical component of the model ensured that parameter values of the study-specific RR were identical across periods in studies with more than one data point (eg, RR for period one and RR for period one plus two combined).

We estimated best-fitting model parameters using Markov Chain Monte Carlo methods. Gibbs sampling was used to draw from posterior distributions. We used non-informative prior distributions for all parameters. We ran four parallel Markov Chain Monte Carlo chains and visually assessed whether chains converged. We report medians and 95% credible intervals from the posterior distributions of the cumulative VE. In the absence of any prior knowledge about the probable shape of waning, we explored several functional forms, including linear, power law, sigmoid, and gamma (appendix p 8). We assessed their goodness of fit using the deviance information criterion (DIC), visual assessment, and biological plausibility.

The best-fitting function of VE by duration of follow-up was used to estimate the iVE by duration of follow-up using a novel approach based on Kaplan-Meier survival estimates. More details about the method, including a derivation of the method, are provided in the appendix (p 1). The iVE at time t, termed 1 – σ(t), is retrieved using the following formula:

$$1-\sigma \left(\text{t}\right)=1-\left(\upsilon \left(\text{t}\right)+\int_{\text{x}=0}^{\text{t}-1}\left(\upsilon \left(\text{t}\right)-\sigma \left(\text{x}\right)\right)\frac{\lambda \left(\text{x}\right)}{\lambda \left(\text{t}\right)}\text{dx}\right)$$

In this formula, iVE as a function of VE at time t is termed 1 – σ(t), all iVEs up until time t are termed 1 – σ(x), the baseline rate or force of infection at time t is termed λ(t), and all baseline rates up until time t are termed λ(*x*). υ denotes the relative rate, but we can only estimate relative risks (θ) with our dataset. Severe rotavirus gastroenteritis is a rare outcome, so we assume that θ is approximately equal to υ.

iVE and VE are identical at time t=0, that is to say 1 – σ(0) = 1 – υ(0). The formula can then be used to iterate over all VEs to retrieve iVEs. If changes in baseline rates are not known, they can be assumed to be similar over time, so that:

$$\frac{\lambda \left(\text{x}\right)}{\lambda \left(\text{t}\right)}=1$$

For each stratum, we reported iVE at standard follow-up times. We calculated empirical p values and credible intervals to investigate differences between strata. We ran a sensitivity analysis to calculate iVE with and without observations from trials with large sample sizes.

Analyses were done using R version 3.4.3^26^ and the rjags package.^27^ Code for the model and conversion method is provided online.^28^

### Head-to-head comparison of efficacy for alternative schedules

To compare the efficacy and waning associated with different rotavirus vaccine schedules, we identified RCTs that directly compared different vaccine schedules head to head and requested more detailed unpublished information from the investigators on the number of case counts and individuals occurring in each week of follow-up after the last dose was administered. In sites with available data, we fitted the same models as in the pooled analysis but without the hierarchical parameters. These models used the same waning functions as in the pooled analysis (appendix, p 6) but with refitted parameters. Again, VE by duration of follow-up was converted to iVE, and we calculated empirical p values and credible intervals to investigate differences between schedules.

### Role of the funding source

The funder of the study had no role in study design, data collection, data analysis, data interpretation, or writing of this report. The corresponding author had full access to all the data in the study and had final responsibility for the decision to submit for publication.

## Results

We included 50 observation points from 50 observations published before April 4, 2018, in populations with low under-5 mortality (15 observations), medium under-5 mortality (11 observations), and high under-5 mortality (24 observations) (table 1). We excluded trials that evaluated special groups, such as populations with high HIV prevalence^46^ and breastfed infants.^47^ We excluded the Finnish Extension Trial^48^, ^49^ because the results could not be disentangled from a pooled estimate for five European countries reported separately.^42^ For the pooled analysis we focused on infant schedules, so excluded the neonatal RotaShield trial^50^ in Ghana and the neonatal schedule group of the RV3-BB trial^15^ in Indonesia. The neonatal schedule group of the RV3-BB trial^15^ was included in a separate head-to-head comparison of the infant and neonatal schedule in Indonesia.

**Table 1:** Observations from published randomised controlled trials included in the pooled analysis of infant schedules

	**Schedule**		**Vaccine brand**	Score*	**Mean follow-up (months since final dose)**	**Non-vaccinated group**†		**Vaccinated group**		**Cumulative efficacy (95% confidence intervals)**
	Mean age	(weeks) at dose 1	Doses			Cases	Number of individuals	Cases	Number of individuals	
**High-mortality countries**										
Bangladesh^9^	10·0	2	Rotarix	V11–20	8·1	35	301	9	292	73% (45 to 87)
Malawi^10^	11·0	2	Rotarix	V11–20	7·7	38	483	21	525	49% (15 to 70)
Malawi^10^	11·0	2	Rotarix	V11–20	15·6	53	483	38	525	34% (2 to 56)
South Africa‡^11^	11·0	2	Rotarix	V11–20	7·7	9	408	5	418	46% (−60 to 82)
South Africa‡^11^	11·0	2	Rotarix	V11–20	15·6	13	408	9	418	32% (−56 to 71)
Malawi^10^	6·2	3	Rotarix	V11–20	7·7	38	483	20	505	50% (15 to 70)
Malawi^10^	6·2	3	Rotarix	V11–20	15·6	53	483	32	505	42% (12 to 62)
South Africa‡^11^	6·2	3	Rotarix	V11–20	7·7	9	408	1	425	89% (16 to 99)
South Africa‡^11^	6·2	3	Rotarix	V11–20	15·6	13	408	2	425	85% (35 to 97)
Bangladesh^12^	8·3	3	RotaTeq	V11–20	8·0	31	565	17	563	45% (2 to 69)
Bangladesh^12^	8·3	3	RotaTeq	V11–20	14·7	56	565	33	563	41% (11 to 61)
Ghana^29^	8·4	3	RotaTeq	V11–20	8·0	42	1081	15	1081	64% (36 to 80)
Ghana^29^	8·4	3	RotaTeq	V11–20	14·5	57	1081	26	1081	54% (28 to 71)
Kenya^29^	7·3	3	RotaTeq	V11–20	8·1	12	611	2	610	83% (26 to 96)
Kenya^29^	7·3	3	RotaTeq	V11–20	12·3	14	611	5	610	64% (1 to 87)
Mali§^29^	6·9	3	RotaTeq	V11–20	8·5	4	921	4	921	0% (−299 to 75)
Mali^29^	6·9	3	RotaTeq	V11–20	14·9	58	921	48	921	17% (−20 to 43)
Niger^14^	6·8	3	ROTASIIL	V11–20	5·6	87	1728	31	1780	65% (48 to 77)
India¶^13^	6·9	3	ROTASIIL	V11–20	8·3	94	3498	61	3527	36% (11 to 53)
India^13^	6·9	3	ROTASIIL	V11–20	20·0	275	3502	171	3533	38% (26 o 49)
India^16^	6·8	3	ROTAVAC	V11–20	8·2	64	2187	56	4354	56% (37 to 69)
India^16^	6·8	3	ROTAVAC	V11–20	13·4	76	2187	71	4354	53% (35 to 66)
Indonesia‖^15^	9·3	3	RV3-BB	V11–20	7·5	17	504	4	511	77% (32 to 92)
Indonesia‖^15^	9·3	3	RV3-BB	V11–20	13·5	28	504	14	511	51% (7 to 74)
**Medium-mortality countries**										
China^30^	9·6	2	Rotarix	V11–20	4·0	32	1573	8	1575	75% (46 to 88)
China^30^	9·6	2	Rotarix	V11–20	16·5	75	1573	21	1575	72% (55 to 83)
Latin America^31^ (n=3)	8·4	2	Rotarix	V11–20	7·5	34	454	27	1392	74% (58 to 84)
Latin America^32^ (n=6)	8·6	2	Rotarix	V11–20	7·9	19	2099	7	4211	82% (56 to 92)
Latin America^33^ (n=10)	8·0	2	Rotarix	V11–20	8·8	58	7081	10	7205	83% (67 to 91)
Latin America^33^ (n=10)	8·0	2	Rotarix	V11–20	20·5	161	7081	32	7205	80% (71 to 87)
China^34^	8·5	3	RotaTeq	V11–20	9·8	52	1946	11	1930	79% (59 to 89)
Latin America^35^ (n=5)	9·7	3	RotaTeq	Hosp/ED	19·0	10	2237	1	2252	90% (22 to 99)
USA^36^ (Navajo)	>6	3	RotaTeq	C11–24	8·8	37	403	4	392	89% (69 to 96)
Vietnam^12^	9·7	3	RotaTeq	V11–20	8·0	7	442	2	446	72% (−36 to 94)
Vietnam^12^	9·7	3	RotaTeq	V11–20	12·3	15	442	5	446	67% (10 to 88)
**Low-mortality countries**										
Europe^37^ (n=6)	11·5	2	Rotarix	V11–20	5·3	60	1302	5	2572	96% (90 to 98)
Europe^37^ (n=6)	11·5	2	Rotarix	V11–20	17·3	127	1302	24	2572	90% (85 to 94)
Finland^38^	8·3	2	Rotarix	V11–20	5·3	5	123	1	245	90% (15 to 99)
Finland^38^	8·3	2	Rotarix	V11–20	17·3	10	123	3	245	85% (46 to 96)
Japan^39^	7·7	2	Rotarix	V11–20	20·6	12	250	2	498	92% (63 to 98)
Southeast Asia^40^ (n=3)	12·0	2	Rotarix	V11–20	7·4	15	5256	0	5263	97% (46 to 100)
Southeast Asia^40^ (n=3)	12·0	2	Rotarix	V11–20	31·7	64	5256	2	5263	97% (87 to 99)
Southeast Asia^40^ (n=3)	12·0	2	Rotarix	V11–20	19·5	51	5256	2	5263	96% (84 to 99)
USA^41^	13·0	2	Rotarix	All RVGE	7·0	18	107	2	108	89% (54 to 97)
Europe^42^ (n=5)	10·0	3	RotaTeq	C17–24	13·3	43	1188	0	1120	99% (80 to 100)
Europe^42^ (n=5)¶	10·0	3	RotaTeq	C17–24	19·0	61	1155	1	1088	98% (87 to 100)
USA^35^	9·7	3	RotaTeq	Hosp/ED	19·0	58	12 179	3	12 284	95% (84 to 98)
Finland and USA^43^	10·0	3	RotaTeq	C17–24	4·4	6	661	0	651	92% (−38 to 100)
Japan^44^	7·6	3	RotaTeq	C17–24	6·7	10	381	0	380	95% (19 to 100)
USA^45^	>8	3	RotaTeq	C17–24	5·5	8	183	0	187	94% (1 to 100)

The cumulative efficacy for reported periods of follow-up after two or three doses of live oral rotavirus vaccines are shown.

* Scores denote the points on the Vesikari scale 11–20 (V11–20) or Clark scale 17–24 (C17–24). All RVGE denotes any severity of rotavirus-positive gastroenteritis. Hosp/ED denotes rotavirus-positive hospitalisation or emergency department visit.

† All randomised controlled trials were placebo controlled with the exception of the Rotarix trial in Bangladesh.

‡ Data only extracted for the South African cohort that was followed for two successive seasons.

§ There were surveillance issues in the first year of trial in Mali that have been postulated to contribute to the low efficacy in the first period, but we did not adjust for this.

¶ N values were adjusted to be the same for both follow-up periods in the Bayesian meta-regression.

‖ For the neonatal schedule, the cumulative efficacy was 94% (95% CI 55–99) after about 9 months of follow-up and 75% (43–89) after about 15 months.

Most of the data points (41 [82%] of 50) were reported using a Vesikari score of 11–20. There were 24 data points for Rotarix, 19 for RotaTeq, two for RV-3BB, three for ROTASIIL, and two for ROTAVAC. More data points (30 [60%] of 50) were based on a three-dose schedule than a two-dose schedule (20 [40%] of 50). The mean age of administration for the first dose ranged from 6 weeks to 13 weeks.

**Figure 1:**
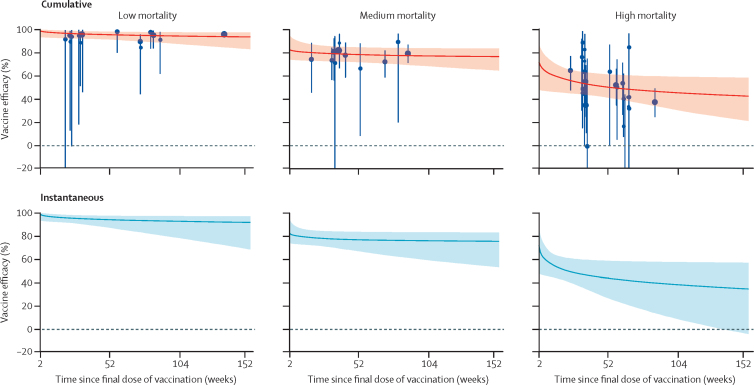
Median and 95% credible intervals of cumulative and instantaneous vaccine efficacy by duration of follow-up and setting after two or three doses of oral rotavirus vaccination (infant schedules only) A simple power function was used to represent vaccine waning over time; equivalent plots based on other potential waning functions are available in the appendix (p 10). Each blue dot represents the VE for each observation. The size of the dot represents the relative sample size of the study. The error bars represent 95% CIs around the VE. VE=vaccine efficacy.

We estimated VE and iVE (median and 95% credible intervals) by duration of follow-up (figure 1, table 2). In settings with low mortality (15 observations), iVE pooled for infant schedules of Rotarix and RotaTeq was 98% (95% credibility interval 93–100) 2 weeks following the final dose of vaccination and 94% (87–98) after 12 months. Equivalent pooled estimates for medium-mortality settings (11 observations) were 82% (74–92) after 2 weeks and 77% (67–84) after 12 months. In settings with high mortality (24 observations), there were five vaccines with observation points for infant schedules. The pooled iVE was 66% (48–81) after 2 weeks of follow-up and 44% (27–59) after 12 months.

**Table 2:** Median instantaneous vaccine efficacy and 95% credible intervals by duration of follow-up and setting after two or three doses of oral rotavirus vaccination (infant schedules only)

	**Low-mortality countries**	**Medium-mortality countries**	**High-mortality countries**	**High-mortality countries (except India)**	**India**
2 weeks	98% (93 to 100)	82% (74 to 92)	66% (48–81)	81% (56–94)	54% (−78 to 88)
1 month	98% (93 to 100)	81% (74 to 90)	62% (47–75)	74% (53–88)	52% (−89 to 88)
2 months	97% (93 to 99)	80% (73 to 87)	57% (45–67)	66% (50–79)	49% (−105 to 87)
3 months	96% (92 to 99)	79% (73 to 86)	54% (44–64)	61% (48–72)	48% (−108 to 86)
6 months	95% (91 to 98)	78% (71 to 84)	49% (40–61)	49% (38–64)	45% (−115 to 86)
9 months	95% (89 to 98)	77% (69 to 84)	46% (33–60)	42% (22–61)	43% (−124 to 86)
12 months	94% (87 to 98)	77% (67 to 84)	44% (27–59)	36% (5–60)	42% (−128 to 85)
18 months	94% (83 to 97)	77% (63 to 84)	41% (17–58)	27% (−26 to 59)	41% (−135 to 85)
24 months	93% (79 to 97)	76% (59 to 83)	38% (9–58)	19% (−54 to 57)	40% (−139 to 85)
36 months	92% (69 to 97)	76% (53 to 83)	35% (−4 to 57)	7% (−107 to 56)	39% (−149 to 85)
48 months	91% (58 to 97)	75% (48 to 83)	32% (−14 to 57)	−2% (−154 to 56)	38% (−154 to 85)
60 months	91% (48 to 97)	75% (44 to 83)	30% (−23 to 57)	−10% (−200 to 55)	37% (−163 to 85)

We found good evidence that iVE was significantly lower in medium-mortality settings than in low-mortality settings after 2 weeks and 12 months of follow-up. The median absolute percentage point difference in iVE was 16% (95% credibility interval 4–24, p=0·0023) at 2 weeks and 17% (8–28, p=0·0022) at 12 months. We found weak evidence that iVE was significantly lower in high-mortality settings compared with medium-mortality settings after 2 weeks and strong evidence that iVE was lower after 12 months. The median difference was 16% (95% credibility interval −1 to 38, p=0·089) at 2 weeks and 33% (15 to 51, p=0·0011) at 12 months.

Two large studies in India (of ROTAVAC^16^ and ROTASIIL)^13^ were included in the high-mortality stratum. Given their large sample sizes, we investigated whether these two studies were driving the results in the high-mortality setting. Therefore, we ran a sensitivity analysis to calculate iVE with and without the Indian data points, and for India alone (table 2; appendix p 27). We found no evidence that iVE significantly differed after 2 weeks or 12 months of follow-up when excluding the Indian data points from the high-mortality stratum. The median difference absolute percentage point difference in iVE was −13% (95% credibility interval −39 to 16, p=0·81) after 2 weeks and 8% (–22 to 42, p=0·31) after 12 months.

A simple power function was fitted in all strata because this required the fewest assumptions and parameters and had goodness of fit (DIC scores) that were consistently favourable across all strata of interest compared with other functions (appendix p 8). Results for alternative functions are shown in the appendix (p 10).

**Figure 2:**
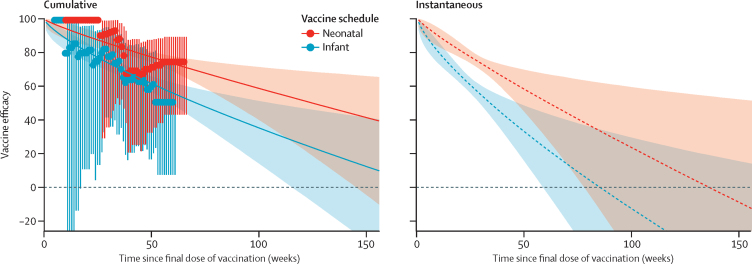
Median and 95% credible intervals of cumulative and instantaneous vaccine efficacy by duration of follow-up and type of schedule (neonatal *vs* infant) following three doses of RV3-BB in Indonesia A simple power function was used to represent vaccine waning over time; equivalent plots based on other potential waning functions are available in the appendix (p 19). Data points shown on the left-hand panel represent observed vaccine efficacies derived from cumulative Kaplan-Meier hazard ratios, and error bars with their corresponding 95% confidence intervals. Solid lines and dashed lines represent medians. Shaded areas represent 95% credible intervals.

We found few RCTs with head-to-head comparisons of different schedules. In Indonesia, a three-dose neonatal RV3-BB schedule (administered at 0–5 days, 8–10 weeks, and 14–16 weeks) was compared with a three-dose RV3-BB infant schedule (administered at 8–10 weeks, 14–16 weeks, and 18–20 weeks).^15^ For the neonatal schedule, the VE was 94% (95% confidence interval 55–99) after about 9 months of follow-up and 75% (43–89) after about 15 months. For the infant schedule, VE was 77% (32–92) after about 8 months and 51% (7–74) after about 14 months (table 1). For this trial, we were able to obtain the number of events in each week of follow-up to better inform estimates of iVE over time. For the neonatal schedule, the estimated iVE was 98% (92–100) after 2 weeks, 77% (73–80) after 6 months, and 57% (42–69) after 12 months of follow-up. For the standard infant schedule, iVE was 95% (89–98) after 2 weeks, 60% (55–64) after 6 months, and 31% (12–48) after 12 months of follow-up (figure 2). We found no significant difference between the two schedules after 2 weeks of follow-up, but strong evidence that the neonatal schedule had higher iVE after 6 months and 12 months of follow-up. The median difference in iVE was 3% (–1 to 10, p=0·088) after 2 weeks of follow-up, 17% (13 to 22, p=0·00049) after 6 months, and 26% (13 to 48, p=0·017) after 12 months.

We used a simple power function for the head-to-head analyses because it required the minimum number of assumptions and parameters and had favourable DIC scores (appendix p 8). Results for alternative functions are also shown in the appendix (p 19).

The only other trial with head-to-head comparison of schedules was a multicountry trial comparing infant schedules of Rotarix in South Africa and Malawi. We were unable to obtain the underlying dataset for this trial. In both countries, a three-dose schedule (administered at 6 weeks, 10 weeks, and 14 weeks) had higher VE than the two-dose schedule (administered at 10 weeks and 14 weeks), but the CIs were wide (table 1).

## Discussion

Our analysis showed that live oral rotavirus vaccines provide high and durable protection in low-mortality and medium-mortality settings. Efficacy is lower and wanes more rapidly in high-mortality settings, but in these settings, more than 60% of rotavirus gastroenteritis hospital admissions occur before age 1 year, and more than 90% occur before age 2 years.^51^ Thus, live oral rotavirus vaccines are still likely to provide substantial benefit in these settings, irrespective of waning.

The reasons for lower rotavirus vaccine efficacy in resource-poor settings are not well understood. Immunogenicity studies have shown lower geometric mean concentrations in resource-poor settings than in high-income settings.^52^ Hypotheses for lower immunogenicity include interference by maternal antibodies, interference by oral polio vaccines, neutralising factors present in breastmilk, malnutrition, other enteric coinfections, rotavirus strain diversity, and HIV infection. Competition in the gut has also been proposed as a reason for the lower performance of oral polio vaccine in resource-poor settings.^53^, ^54^, ^55^ Research is underway to assess the role of maternal antibodies and gut microbiota in the immune response to rotavirus vaccines in British, Malawian, and Indian infants.^56^ Two pivotal cohort studies from Mexico^57^ and India^58^ have reported contrasting estimates of the protection conferred by natural infections against subsequent disease. In Mexico (a medium-mortality setting), two previous infections (asymptomatic or symptomatic) conferred 100% protection against subsequent moderate or severe rotavirus gastroenteritis. In India (a high-mortality setting), the equivalent protection was 57% after two previous infections (and 79% after three previous infections). If natural infections are less likely to protect against moderate and severe rotavirus gastroenteritis in higher-mortality settings than in lower-mortality settings, then a live oral vaccine mimicking natural infection might also have lower estimated efficacy in children in these settings.

The reported declines in instantaneous efficacy might not be entirely caused by declining vaccine-induced antibodies. Some of the decrease could be explained by a higher incidence of natural asymptomatic and mild infections (and thus preferential immune boosting) among unvaccinated controls compared with vaccine recipients. In these circumstances the risk of severe rotavirus gastroenteritis in vaccine recipients would gradually converge with, and might exceed, the risk in unvaccinated controls over time. This phenomenon has been described previously in the context of so-called leaky vaccines.^59^ Our analysis of the infant and neonatal schedules for RV3-BB in Indonesia suggested a positive protective effect of the vaccine in the first 18 months of follow-up, but extrapolation of the curves suggested a negative effect thereafter. This would be consistent with preferential natural boosting among non-vaccine recipients but is speculative, because it involves extrapolating beyond the observed period of follow-up in the trial. Reanalysis of data from an RCT in Bangladesh^60^ has allowed these effects to be partly disentangled by excluding any children who had an episode of non-severe rotavirus gastroenteritis. This finding explained some, but not all, of the reduction in vaccine efficacy over time. However, it was not possible to exclude infants who had previous asymptomatic infections, which might also have an important role.

Head-to-head comparisons of schedules for the same vaccine were rare, and more evidence is needed from more places on the relative benefits of one schedule over another. In our analysis of RV3-BB in Indonesia, the neonatal schedule provided more durable protection than the infant schedule, but this analysis was based on few case counts in each week of follow-up. A neonatal schedule is also likely to result in higher and earlier coverage and fewer vaccine-related intussusception events than an infant schedule, so warrants serious consideration. Other strategies that could help to improve the effect of the vaccines include administering a booster dose later in infancy^17^, ^61^ or using injectable non-replicating vaccines,^62^ but more evidence is needed on the safety and clinical efficacy of both of these options.

For the pooled analysis, we combined evidence for different vaccine products and different infant schedules to avoid having small numbers of data points in each stratum. None of the RCTs compared different brands of rotavirus vaccination head to head in the same population. There were several observations for RotaTeq and Rotarix, but the Rotarix sites included data points from South Africa, which had higher efficacy and lower child mortality relative to the sites evaluated in the RotaTeq trials. Thus, in the absence of head-to-head comparisons from the same trial populations, there is insufficient evidence to favour one product over another in terms of vaccine efficacy and duration of protection. However, the postlicensure experience of countries that have used both Rotarix and RotaTeq does not suggest any material difference in vaccine effect.^63^

Most of the data points were reported against the Vesikari 11–20 scale, but some were reported against the Clark 16–24 scale. These two scores correlate poorly with one another when estimating the proportion of rotavirus gastroenteritis episodes defined as severe.^64^, ^65^ However, this bias is unlikely to change the conclusion that protection is high and durable in the low-mortality stratum, in which the Clark scale was more commonly used.

We stratified our results by mortality and presented pooled results with and without data points from potentially influential studies. We restricted the analysis to RCTs because they represent the gold standard approach for measuring per-protocol vaccine efficacy and provide accurate information about the mean duration of follow-up. Other designs, such as case-control studies, do not permit precise estimation of the mean duration of follow-up. Some case-control studies report vaccine effectiveness by age band, and thus could potentially be used to derive the duration of follow-up, but this approach becomes increasingly crude as the width of the age band increases. Case-control studies are also at risk of bias because infants who have been vaccinated might be different from those who are unvaccinated for both known and unknown reasons. We also restricted the analysis to per protocol rather than by intention to treat because this analysis provided a consistent basis for pooling the different RCTs, ensuring that all infants received the recommended number of doses and that a more consistent starting point was used for the measurement of follow-up. Accurate estimates of iVE following a single dose of rotavirus vaccination would be useful for informing the potential effects of different schedules, but typically there are few infants who receive only a single dose, and even fewer of those infants are followed up for the full duration of the trial.

We reported the initial peak efficacy starting at 2 weeks of follow-up because of uncertainty around the time that antibodies might take to develop after vaccination. In addition, we had to extrapolate our fitted estimates of VE to periods without empirical data (eg, beyond 2 years of follow-up). The absence of empirical data from RCTs is represented by larger credible intervals in these periods. However, this makes comparison of different waning functions difficult. Evidence from RCTs with a longer duration of follow-up or high-quality observational studies is needed to overcome this knowledge gap. A so-called no-waning model required only one parameter, and for this reason had favourable DIC scores in each of the pooled analyses. However, in Indonesia, where all data points were from the same trial, the DIC score was unfavourable. We considered no waning (or no change in RR) to be implausible, given that VE was shown to decrease in nearly all RCTs with more than one follow-up point. However, more studies with multiple follow-up times would be needed to have greater certainty about the appropriate form of vaccine waning.

We used a novel approach to convert estimates from cumulative VE to iVE. We show that this method is able to retrieve iVE, and that standard estimates of cumulative VE might overestimate iVE in the presence of waning. However, there are some limitations in applying this method to the meta-regression used in this study. First, it would be better to use relative rates than RR. We had to compute relative risks because most of the RCTs only reported observed numbers of cases and individuals at a limited number of follow-up times. However, because severe rotavirus gastroenteritis is a relatively rare outcome, risk ratios and rate ratios are expected to be similar, and we assume that this bias is negligible in our study. Second, waning of vaccine efficacy (or conversely, waxing of the relative rate) might interact with changes in baseline rates. This effect would be relatively small on the estimated VE, but might be pronounced when converted to an iVE. Because we had no information on changes in the baseline rates in our studies, we assumed that this rate was constant over time (an assumption that is often made in survival analyses) and did not correct for it. The bias is likely to be in the direction of increasing VE because baseline rates decline with age, particularly in high-mortality settings. Our method should ideally be extended to control for different changing baseline rates in different studies, as would be the case in a pooled analysis. However, even uncorrected iVE should still be a better approximation to true iVE than cumulative VE in the presence of waning.

Because the two Indian studies had larger sample sizes than other studies in the high-mortality stratum, we did a sensitivity analysis to see whether these studies significantly influenced our results. Exclusion of these studies did not alter our findings significantly. Moreover, an analysis in which we estimated iVE for India alone did not provide meaningful results, because there were only four observations, which is too few to generate a reliable pooled estimate in our analysis.

Reviews of the efficacy, effectiveness, and effects of rotavirus vaccines^19^, ^66^, ^67^ have described variation in rotavirus vaccine effects according to under-5 mortality and geographical region. However, to our knowledge, our study is the first to synthesise all the available RCT evidence and to obtain robust estimates of iVE by duration of follow-up. Our study should provide important evidence for estimating and monitoring the effects of rotavirus vaccines in different settings. Our analysis provides the most comprehensive evidence to date that rotavirus vaccine efficacy is lower and wanes more rapidly in high-mortality settings than in low-mortality settings. The earlier peak age of disease in these settings means that live oral rotavirus vaccines are still likely to provide substantial benefit, but strategies with the potential to further increase the effects of vaccines, such as neonatal vaccination, warrant serious consideration. Monitoring the age distribution of rotavirus disease cases in the years following vaccine introduction will also be important. Consistent with the basic theory of infectious disease dynamics, a reduction in the incidence of infection (eg, from vaccination) should lead to an increase in the mean age of infection. As more children become infected at older ages, the need for more durable rotavirus vaccines might become more pressing.
