# Supplementary material for: Efficacy of live oral rotavirus vaccines by duration of follow-up: a meta-regression of randomised controlled trials
Source: Lancet Infect Dis. 2019 Jul;19(7):717–27. doi: 10.1016/S1473-3099(19)30126-4 (PMC6595176; doi:10.1016/S1473-3099(19)30126-4)
Supplement: Supplementary appendix [file mmc1.pdf]

THE LANCET Infectious Diseases

Supplementary webappendix

This webappendix formed part of the original submission and has been peer reviewed. We post it as supplied by the authors.

Supplement to: Clark A, van Zandvoort K, Flasche S, et al. Efficacy of live oral rotavirus vaccines by duration of follow-up: a meta-regression of randomised controlled trials. *Lancet Infect Dis* 2019; published online June 6. http://dx.doi.org/10.1016/S1473-3099(19)30126-4.

Efficacy of live oral rotavirus vaccines by duration of follow-up: a meta-regression of randomised controlled trials

Appendix

Contents

			Page
**1**		**Method for approximating instantaneous vaccine efficacy (iVE) from cumulative vaccine efficacy (VE)**	**1**
**2**		**Equations of the alternative waning functions evaluated**	**3**
	2.1	*Pooled analysis – meta-regression*	3
	2.2	*Head-to-head trial: analysis of RV3-BB Indonesia trial*	6
**3**		**Comparison of alternative waning functions**	**8**
	3.1	*Deviance Information Criteria (DIC)*	8
	3.2	*Visual comparison*	8
	3.2.1	*Visual comparison of alternative waning functions in meta-regression (pooled analysis)*	10
	3.2.2	*Visual comparison of alternative waning functions in head-to-head trial (RV3-BB Indonesia)*	19
**4**		**Sensitivity analysis for the high-mortality stratum**	**27**
**5**		**References**	**27**

1. Method for approximating instantaneous vaccine efficacy (iVE) from cumulative vaccine efficacy (VE)

Vaccine efficacy is commonly defined as the reduction in the attack rate in vaccinated compared to unvaccinated clinical trial participants, and estimated as 1-*RR*, where RR is the risk-ratio or rate-ratio. If the RR is constant over the period of follow-up (i.e. the time since administration of the final dose in the vaccination schedule), then 1-*RR* is a reliable indicator of the cumulative vaccine efficacy (VE) over the entire period of follow-up as well as the instantaneous vaccine efficacy (iVE) at specific follow-up times within that period. However, temporal changes in RR may occur during the period of follow-up. Under these circumstances, iVE may differ from VE.

Analogue to the Kaplan Meier estimands, the probability of not having encountered an infection or disease episode up to time *t* is:

$$S(t) = \prod_{x=0}^{t} 1 - \lambda(x)\sigma(x)$$

where $\lambda(x)$ is the force of infection at time x and $\sigma(x)$ is the vaccine effect expressed as the instantaneous rate-ratio at time x. Hence, VE can be estimated via the respective relative rate, ϑ, as:

$$1 - \vartheta(t) = 1 - \frac{-\log \prod_{x=0}^{t} 1 - \lambda(x)\sigma(x)}{-\log \prod_{x=0}^{t} 1 - \lambda(x)}$$

As the daily force of infection is usually very small ($\lambda(x) \ll 1$), the Nelson-Aalen estimator can be used to approximate VE(t):

$$1 - \vartheta(t) \approx 1 - \frac{\int_{x=0}^{t} \lambda(x)\sigma(x)dx}{\int_{x=0}^{t} \lambda(x)dx}$$

We assume that $\lambda(x)\sigma(x)$ and $\lambda(x)$ are well approximated piecewise linear functions, i.e.

$$1 - \vartheta(t) \approx 1 - \frac{\int_{x=0}^{t-1} \lambda(x)\sigma(x)dx + \lambda(t)\sigma(t)}{\int_{x=0}^{t-1} \lambda(x)dx + \lambda(t)}$$

This can be rewritten as:

$$iVE(t) \approx 1 - \sigma(t) \approx 1 - \left(\vartheta(t) + \int_{x=0}^{t-1} (\vartheta(t) - \sigma(x))\frac{\lambda(x)}{\lambda(t)}dx\right)$$

If discretised this provides a recursive equation to calculate iVE(t), starting at $\sigma(0)=\vartheta(0)$. Note that the final term, $\frac{\lambda(x)}{\lambda(t)}$, is the ratio between the force-of-infection at time x and that at time t. Therefore, this

method requires all values of VE and all forces of infection up until time t, in order to successfully convert to iVE.

As the actual force of infection is usually unknown, one can assume no seasonality in the force of infection and set this term to 1. This assumption is commonly made in other analyses of VE, such as when using a Cox or Poisson regression.

Note that this method transforms the VE as derived using a rate-ratio to iVE. While this is appropriate for leaky vaccines, the VE of an all-or-nothing vaccine should be estimated using a relative risk instead [1]. As risks and rates are numerically similar when the outcome of interest is rare, the appropriateness of this method for all-or-nothing vaccine will depend on the prevalence of the outcome. Similarly, as seasonal effects in the force of infection are not always known, strong seasonal forcing may bias estimates of iVE.

Figure 1 shows the difference between VE and iVE. The left panel shows data simulated for a hypothetical scenario using a simple Susceptible-Infected model. This assumes a leaky vaccine with initial vaccine efficacy of 75%, no seasonal effects, relatively rapid waning (half-life of protective antibodies = 800 days) and a disease incidence rate of 200 per 100 000 person-days. As illustrated by the overlap of the black and yellow lines, the equation method described above correctly recovered the simulated iVE. The right panel shows an example for rotavirus based on the data presented for the high-mortality stratum in the main paper. Here, the true iVE is unknown, but estimated from the fitted values of VE.

Note that in both scenarios, VE overestimates iVE. This is because VE at time t can be seen as an average over all iVE values up until time t, in contrast to the iVE value at time t. The equation method is able to retrieve the iVE irrespective of the waning function used. Note: in the left panel a sigmoidal waning function is used and in the right panel a power waning function is used.

Figure 1. Cumulative and instantaneous vaccine efficacy by time since vaccination for a hypothetical vaccination scenario and for a rotavirus example based on trial data from high mortality settings.

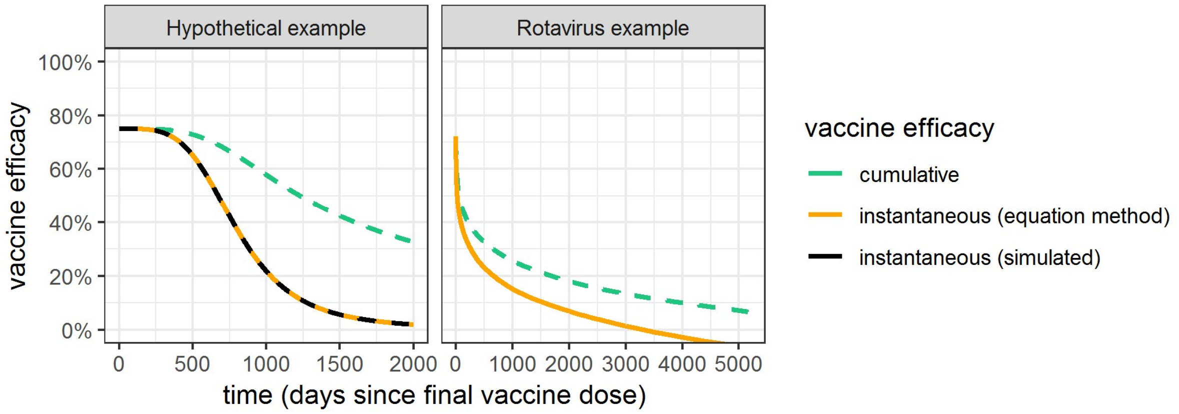

2. Equations of the alternative waning functions evaluated

We used two analyses in our study, a meta-regression to generate pooled estimates of vaccine efficacy, and a Poisson regression in which we investigated waning of an individual trial where we had weekly-follow up data. Different waning functions were compared visually, statistically (comparing the Deviance Information Criteria), and were assessed for biological plausibility. Fitted curves representing VE over time were converted to iVE using the method outlined above.

2.1. Pooled analysis – meta-regression

We fitted curves estimating overall cumulative vaccine efficacy to the observed cumulative vaccine efficacies. We performed a Poisson regression using a hierarchical Bayesian model, where study-specific relative risks were centred around an overall latent relative risk (RR) in each respective mortality stratum.

We investigated multiple waning functions. In functions that are listed as bounded, the RR is bounded between 0 and 1. As a result, *cumulative* vaccine efficacy in these functions is bounded between 100% and 0%. Note that this does not necessarily bound the *instantaneous* vaccine efficacy, as a *cumulative* vaccine efficacy above 0% can still result from negative *instantaneous* vaccine efficacies in a certain period.

Table 1 below illustrates the functional form of the natural logarithm of the latent time-specific RR as fitted in each respective waning function in the meta-regression. Time specific cumulative vaccine efficacy was then estimated as $1 - e^{\log(\theta)}$. Note that the entire waning function is used in computing the RR, and that individual parameters in each waning function are not easily interpretable. Each function has up to 4 different parameters, depending on the complexity of the waning function: ϕ, t, α, and β. The parameter ϕ was estimated for each study separately as ϕ_i (centred around an overall latent ϕ, whose prior was assigned mean 0 and standard deviation equal to the square root of the estimated between-study variance), whereas α and β were the same across studies. t is the time-component of the function and refers to the months since final dose of vaccination was administered.

We used non-informative prior distributions for all parameters. Parameters were all assigned a prior following a normal distribution with mean 0 and a precision of 0.01 (standard deviation of 10). ϕ acted as a hyperprior for ϕ_i. The prior for the latter followed a normal distribution with a mean of ϕ, whilst we used a uniform distribution between 0 and 2 for its standard deviation.

The high-mortality stratum was modelled three times. Once including all countries, once excluding India, and once for India only. Note that, because the analysis for India only included two studies, the

hierarchical construct of the analysis over-inflated uncertainty around our estimates. There were only four observations, which is too few to generate a reliable pooled estimate in our analysis.

Table 1. Waning functions for alternative models

**Model**	**Log relative risk**
No waning	$\log\theta(t) = \phi$
Linear	$\log\theta(t) = \phi + \alpha + \log(t)$
Power	$\log\theta(t) = \phi + e^{\alpha}\log(t)$
Power 2	$\log\theta(t) = \phi + \alpha\log(t)$
Power 3	$\log\theta(t) = \phi + \alpha\, t$
Power (bounded)	$\log\theta(t) = \alpha + e^{\beta}\log(t) - \log(e^{\phi} + e^{\alpha}t^{e^{\beta}})$
Sigmoid (bounded)	$\log\theta(t) = \phi - \log(\mathrm{e}^{\phi} + \mathrm{e}^{\alpha}\mathrm{e}^{-\mathrm{e}^{\beta}t})$
Gamma (bounded)*	$\log\theta(t) = \log(\Gamma(\mathrm{t}, e^{\phi}, e^{\alpha}))$

* Γ refers to the cumulative gamma function

The median and 95% credible interval of the marginal posterior distribution for each fitted parameter is given in table 2.

Table 2. Posterior estimates of parameters in each alternative model, stratified by mortality stratum

Mortality stratum	Model	ϕ	α	β
Low mortality	No waning	-3.05 (-3.91 — -2.44)	.	.
	Linear	-3.86 (-6.12 — -2.72)	-6.6 (-9.34 — -5.69)	.
	Power	-3.68 (-6.04 — -2.65)	-1.55 (-6.72 — 0.05)	.
	Power 2	-4.55 (-6.71 — -2.77)	0.55 (-0.06 — 1.25)	.
	Power 3	-3.72 (-5.29 — -2.67)	0.04 (-0.01 — 0.11)	.
	Power (bounded)	2.48 (-2.46 — 8.37)	-1.66 (-6.13 — 4.08)	-1.14 (-6.39 — 0.16)
	Sigmoid (bounded)	-2.41 (-9.56 — 4.65)	1.15 (-5.96 — 8.09)	-3.51 (-7.38 — -2.28)
	Gamma (bounded)	-0.11 (-0.81 — 0.66)	-6.12 (-9.32 — -4.08)	.
Medium mortality	No waning	-1.54 (-1.86 — -1.27)	.	.
	Linear	-1.76 (-2.81 — -1.35)	-5.78 (-9.31 — -4.54)	.
	Power	-1.71 (-2.79 — -1.34)	-2.94 (-7.34 — -0.75)	.
	Power 2	-1.44 (-2.98 — -0.57)	-0.05 (-0.41 — 0.55)	.
	Power 3	-1.56 (-2.24 — -1.11)	0 (-0.03 — 0.05)	.
	Power (bounded)	-1.92 (-5.07 — 1.87)	-3.45 (-6.72 — 0.33)	-2.95 (-7.48 — -0.9)
	Sigmoid (bounded)	-1.46 (-6.87 — 1.36)	0.01 (-5.35 — 2.79)	-4.72 (-8.6 — -3.06)
	Gamma (bounded)	-1.11 (-1.7 — -0.46)	-7.34 (-10.81 — -5)	.
High mortality	No waning	-0.74 (-1 — -0.54)	.	.
	Linear	-1.1 (-1.72 — -0.7)	-4.37 (-6.95 — -3.66)	.
	Power	-1.18 (-1.98 — -0.68)	-1.67 (-5.83 — -0.67)	.
	Power 2	-1.32 (-1.99 — -0.72)	0.26 (0 — 0.52)	.
	Power 3	-0.98 (-1.26 — -0.58)	0.02 (-0.01 — 0.04)	.
	Power (bounded)	0.73 (-4.02 — 3.2)	-0.7 (-5.03 — 2.05)	-0.73 (-4.73 — 0.05)
	Sigmoid (bounded)	1.75 (-2.25 — 6.53)	2.14 (-1.71 — 6.9)	-3.37 (-7.35 — -2.39)
	Gamma (bounded)	-1.1 (-1.9 — -0.42)	-4.78 (-7.31 — -3.46)	.
High mortality (no India)	No waning	-0.79 (-1.12 — -0.53)	.	.
	Linear	-2.48 (-15.57 — -1.01)	-3.35 (-4.67 — -3.01)	.
	Power	-1.59 (-2.59 — -0.83)	-1.05 (-3.39 — -0.28)	.
	Power 2	-1.77 (-3.43 — -0.73)	0.42 (-0.01 — 1.1)	.
	Power 3	-1.33 (-1.81 — -0.76)	0.05 (0 — 0.09)	.
	Power (bounded)	1.39 (-1.74 — 6.6)	-0.37 (-4.12 — 3.9)	-0.21 (-3.64 — 0.51)
	Sigmoid (bounded)	-1.74 (-6.58 — 3.06)	-0.88 (-5.7 — 3.98)	-2.58 (-5.88 — -1.77)
	Gamma (bounded)	-0.8 (-1.79 — 0.22)	-4.11 (-6.88 — -2.46)	.
India	No waning	-0.61 (-1.96 — 0.76)	.	.
	Linear	-0.79 (-2.29 — 0.61)	-5.35 (-9.04 — -3.82)	.
	Power	-0.73 (-2.2 — 0.56)	-3.48 (-8.1 — -1.53)	.
	Power 2	-0.5 (-2.04 — 1.17)	-0.04 (-0.45 — 0.31)	.
	Power 3	-0.62 (-2.01 — 0.88)	0 (-0.03 — 0.03)	.
	Power (bounded)	0.37 (-4.25 — 6.14)	0.06 (-4.22 — 5.84)	-2.51 (-7.39 — -0.37)
	Sigmoid (bounded)	-0.29 (-3.83 — 4.44)	-0.28 (-3.54 — 4.27)	-4.32 (-8.26 — -2.7)
	Gamma (bounded)	-1.52 (-3.08 — 0.04)	-5.61 (-8.89 — -3.54)	.

Marginal posterior values of each given parameter. Values reported are the median and 95% credible intervals. Cells are empty if a parameter was not included in the specific model.

2.2. Head-to-head trial: analysis of RV3-BB Indonesia trial

We used the same functions (with the exception of 'No waning', which was purely for illustrative purposes in the meta-regression) to look at data from a single trial in Indonesia (belonging to the high under 5 mortality stratum). The methodology and results from this trial are published elsewhere [2]. For this study, we obtained weekly follow-up data from three arms: a placebo group, a group vaccinated using a neonatal schedule (0-5 days, 8-10 weeks, 14-16 weeks) and a group vaccinated using an infant schedule (8-10 weeks, 14-16 weeks, 18-20 weeks). We used this data to construct cumulative hazard estimates and used the same fitting approach as in our meta-regression, fitting to these cumulative estimates. The functional form of the natural logarithms of each fitted RR is illustrated in the table below. They differ from those used in the meta-regression only in that we added a parameter ρ which adjusts for potential additional differences between the two vaccinated schedules. Coefficient ρ was included when estimating vaccine-efficacy for the infant schedule but omitted for the neonatal schedule, and is therefore denoted as ρ_i in table 3. Similarly, the parameters in the waning function, α_i, and β_i, were estimated separately and independent from one another in each respective schedule. Note that ϕ was fitted as the same value in both schedules, but that ρ_i adjusts for any difference in ϕ in the infant schedule. The functions used for the alternative models are given in Table 3.

Table 3. Waning functions for alternative models in the Indonesia trial

**Model**	**Log relative risk**
Linear	$\log\theta_i(t) = \phi + \alpha_i + \log(t) + \rho_i$
Power	$\log\theta_i(t) = \phi + e^{\alpha_i}\log(t) + \rho_i$
Power 2	$\log\theta_i(t) = \phi + \alpha_i \log(t) + \rho_i$
Power 3	$\log\theta_i(t) = \phi + \alpha_i\, t + \rho_i$
Power (bounded)	$\log\theta_i(t) = \alpha_i + e^{\beta_i}\log(t) - \log(e^{\phi} + e^{\alpha_i} t^{e^{\beta_i}}) + \rho_i$
Sigmoid (bounded)	$\log\theta_i(t) = \phi - \log(e^{\phi} + e^{\alpha_i} e^{-e^{\beta_i} t}) + \rho_i$
Gamma (bounded)[i]	$\log\theta_i(t) = \log(\Gamma(t, e^{\alpha_i}, e^{\beta_i})) + \rho_i$

i. Γ refers to the cumulative gamma function

The values (median and 95% credible interval) of the marginal posterior distribution of each fitted parameter are given in Table 4.

Table 4. Posterior estimates of parameters in each alternative model in the Indonesia trial

Stratum	Model	ϕ^i	$\rho_i{}^i$	α_i	β_i
Neonatal schedule	Linear	-2.39 (-5.38 — -0.45)		-2.93 (-4.92 — 0.05)	.
	Power	-6.72 (-8.29 — -5.24)		0.29 (-0.04 — 0.55)	.
	Power 2	-6.21 (-8.29 — -5.13)		1.22 (0.95 — 1.77)	.
	Power 3	-3.11 (-3.68 — -2.58)		0.03 (0.02 — 0.04)	.
	Power (bounded)	3.77 (0.44 — 6.64)		-4.88 (-10.22 — -2.39)	0.67 (0.31 — 0.99)
	Sigmoid (bounded)	-5.91 (-9.26 — 0.33)		-2.53 (-5.88 — 3.85)	-3.13 (-3.47 — -2.84)
	Gamma (bounded)	.		4.34 (4.22 — 4.49)	-7.23 (-7.92 — -6.72)
Infant schedule	Linear	-2.39 (-5.38 — -0.45)	0.57 (-1.92 — 2.43)	-2.22 (-5.04 — -0.36)	.
	Power	-6.72 (-8.29 — -5.24)	2.01 (0.16 — 3.89)	0 (-0.36 — 0.27)	.
	Power 2	-6.21 (-8.29 — -5.13)	1.47 (-0.84 — 3.49)	1.06 (0.83 — 1.38)	.
	Power 3	-3.11 (-3.68 — -2.58)	1.04 (0.36 — 1.74)	0.03 (0.02 — 0.03)	.
	Power (bounded)	3.77 (0.44 — 6.64)	0.29 (-0.64 — 2.32)	-2.45 (-6.79 — 0.66)	0.27 (-0.23 — 1.07)
	Sigmoid (bounded)	-5.91 (-9.26 — 0.33)	-0.4 (-0.72 — 2.41)	-3.5 (-6.98 — 2.96)	-2.74 (-3.63 — -2.15)
	Gamma (bounded)	.	-0.35 (-0.72 — 1.54)	3.59 (3.13 — 4.98)	-6.61 (-8.67 — -5.29)

Marginal posterior values of each given parameter. Values reported are the median and 95% credible intervals. Cells are empty if a parameter was not included in the specific model.

i. Note that values for sigma are the same in the neonatal and infant schedule, whereas ρ_i is only included in the infant schedule. The values for α_i and β_i were fitted separately for both schedules.

3. Comparison of alternative waning functions

3.1 Deviance Information Criteria (DIC)

The table below gives the DIC value for each respective function in each respective stratum. DIC values should be compared relative to all values in each column. Although the 'no waning' function had generally good DIC values in the pooled analysis, it performed the worst in the Indonesia analysis. Similarly, waning Rotavirus vaccine efficacy is broadly accepted. Although we included this function for comparability, we determined it to be biologically unlikely.

Table 5. DIC values for each waning function in each analysis

Function	Low mortality	Medium mortality	High mortality	High mortality (no India)	India	Indonesia
No waning	154.2	133.5	314.0	250.6	63.4	958.9
Linear	154.9	134.6	315.4	252.9	64.5	911.6
Power	152.6	133.8	315.6	250.5	63.8	914.2
Power 2	152.6	134.8	315.9	250.8	65.4	913.6
Power 3	153.0	135.0	314.9	250.5	65.4	921.5
Power (bounded)	153.2	134.0	315.0	251.2	63.9	912.6
Sigmoid (bounded)	153.1	134.3	314.9	250.8	64.1	920.7
Gamma (bounded)	152.0	137.3	315.0	249.8	65.2	913.7

3.2 Visual comparison

The plots below show, for each function, the median estimated waning of instantaneous vaccine efficacy by mortality strata following 2/3 doses of oral rotavirus vaccination (infant schedules only). The full set of plots for all other functions can be found in sections 3.2.1 (pooled analysis) and 3.2.2 (Indonesia analysis).

Figure 2. Median estimated waning of instantaneous vaccine efficacy by mortality strata, for each waning function explored

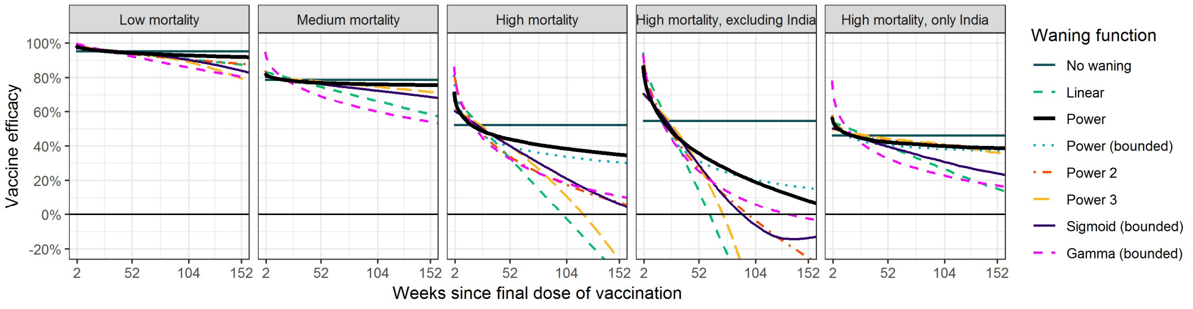

The simple power function (Power) was used in the main paper because it had few parameters and goodness of fit (DIC scores) that were consistently favourable across all strata, compared to the other functions considered. The gamma (bounded) would be a reasonable alternative to use in high mortality countries but had the worst fit in the medium mortality stratum.

3.2.1 Visual comparison of alternative waning functions in meta-regression (pooled analysis)

The next pages show plots with the fitted VE and their corresponding iVE by weeks since final dose of vaccination for alternative waning functions. They correspond to Figure 1 in the main paper of this publication.

Each figure shows the median and 95% credible intervals of cumulative and instantaneous vaccine efcacy by duration of follow-up and setting after two or three doses of oral rotavirus vaccination (infant schedules only). *Each black dot represents the VE for each observation. The size of the dot represents the relative sample size of the study. The error bars represent 95% confidence intervals around the VE.*

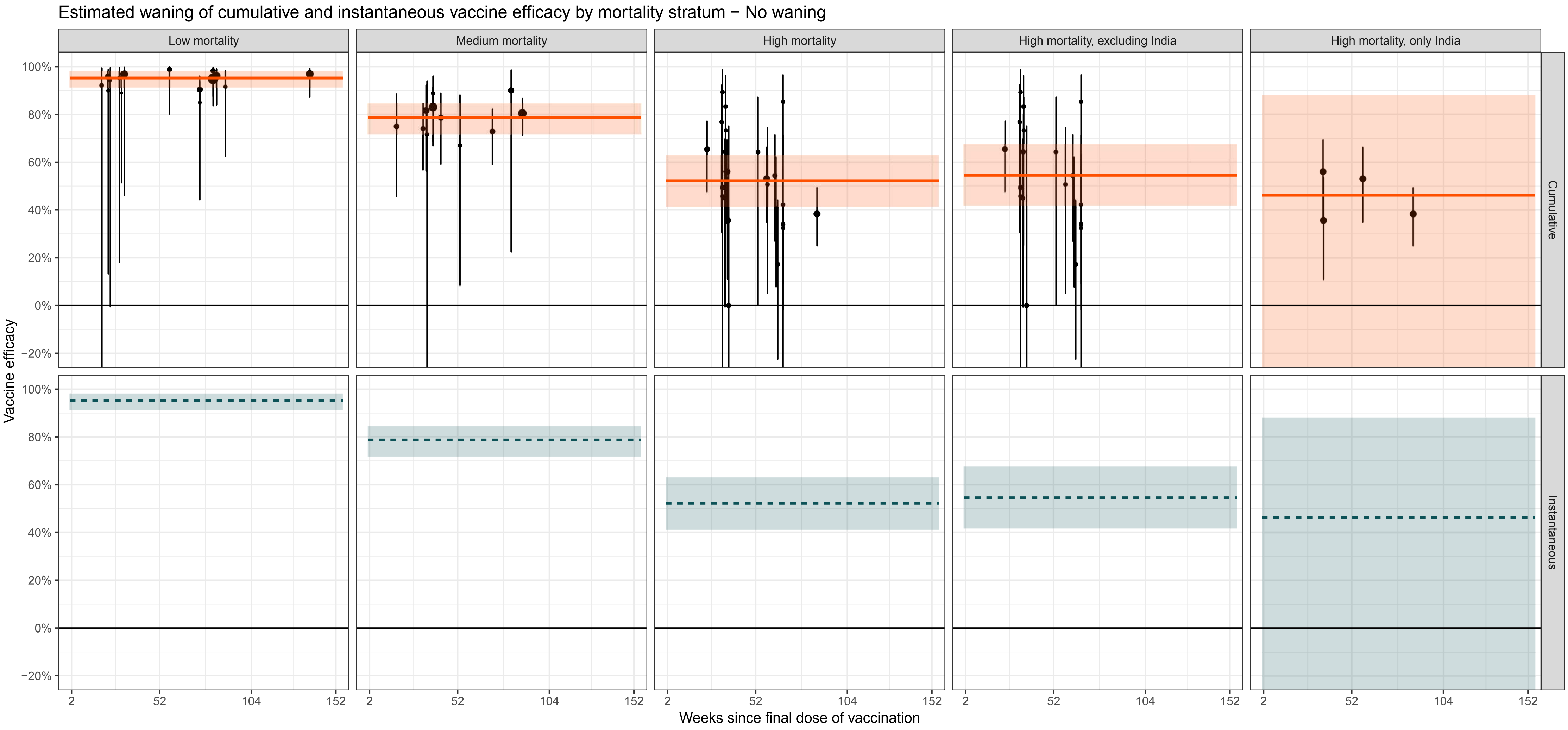

Estimated waning of cumulative and instantaneous vaccine efficacy by mortality stratum − No waning
Low mortality
Medium mortality
High mortality
High mortality, excluding India
High mortality, only India
Cumulative
Instantaneous
Vaccine efficacy
100%
80%
60%
40%
20%
0%
−20%
2
52
104
152
Weeks since final dose of vaccination

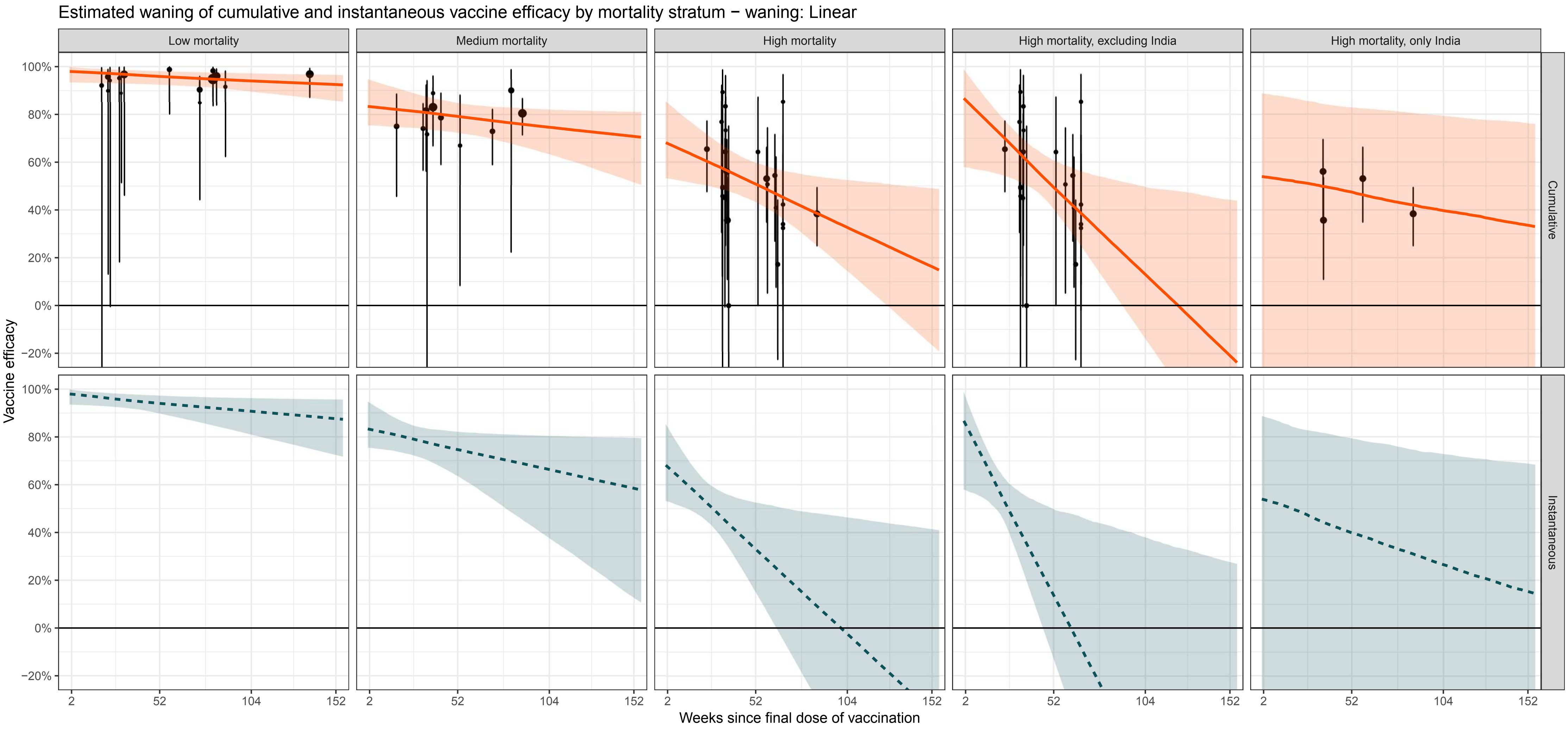

Estimated waning of cumulative and instantaneous vaccine efficacy by mortality stratum − waning: Linear
Low mortality
Medium mortality
High mortality
High mortality, excluding India
High mortality, only India
Cumulative
Instantaneous
Vaccine efficacy
100%
80%
60%
40%
20%
0%
−20%
2
52
104
152
Weeks since final dose of vaccination

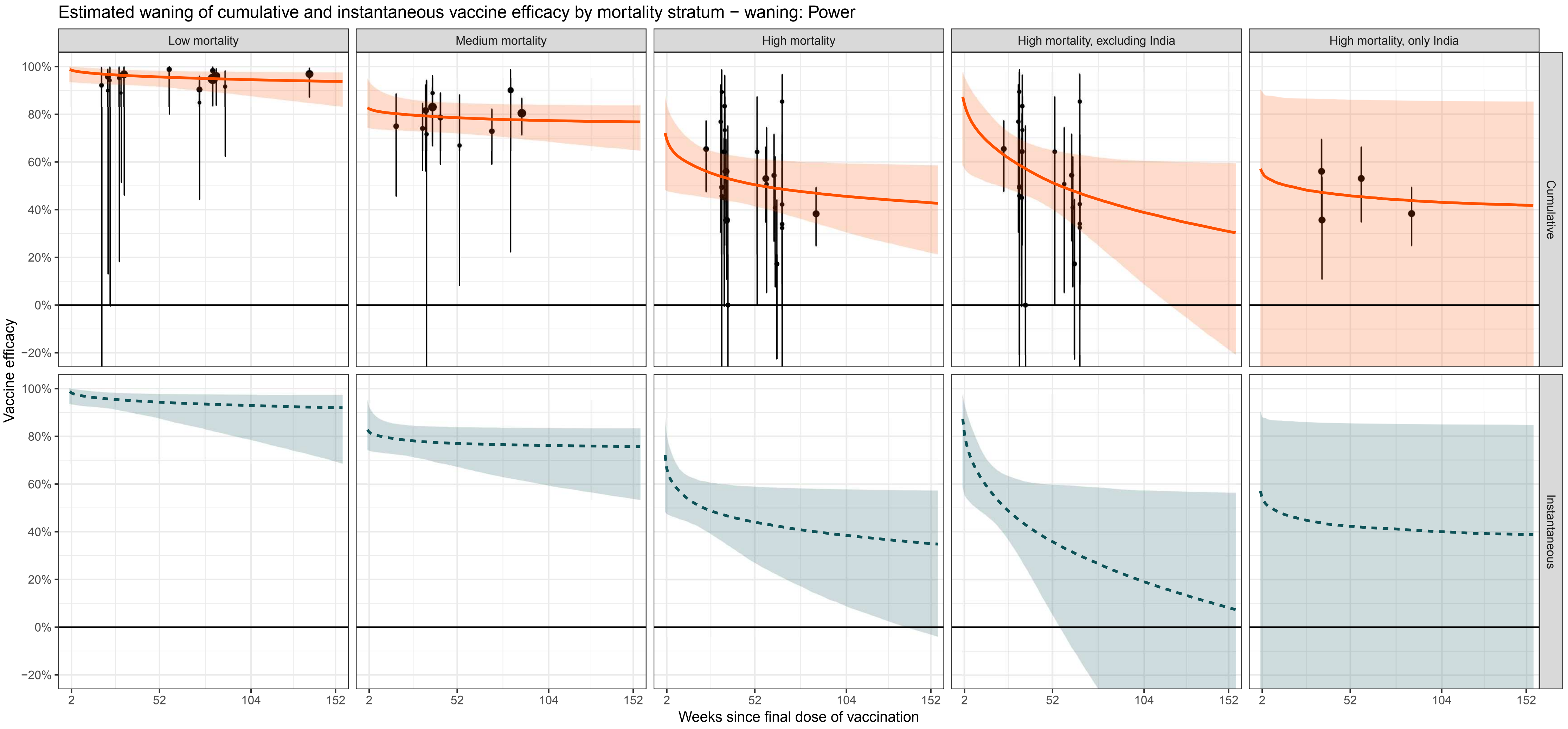

Estimated waning of cumulative and instantaneous vaccine efficacy by mortality stratum − waning: Power
Low mortality
Medium mortality
High mortality
High mortality, excluding India
High mortality, only India
Cumulative
Instantaneous
Vaccine efficacy
100%
80%
60%
40%
20%
0%
−20%
2
52
104
152
Weeks since final dose of vaccination

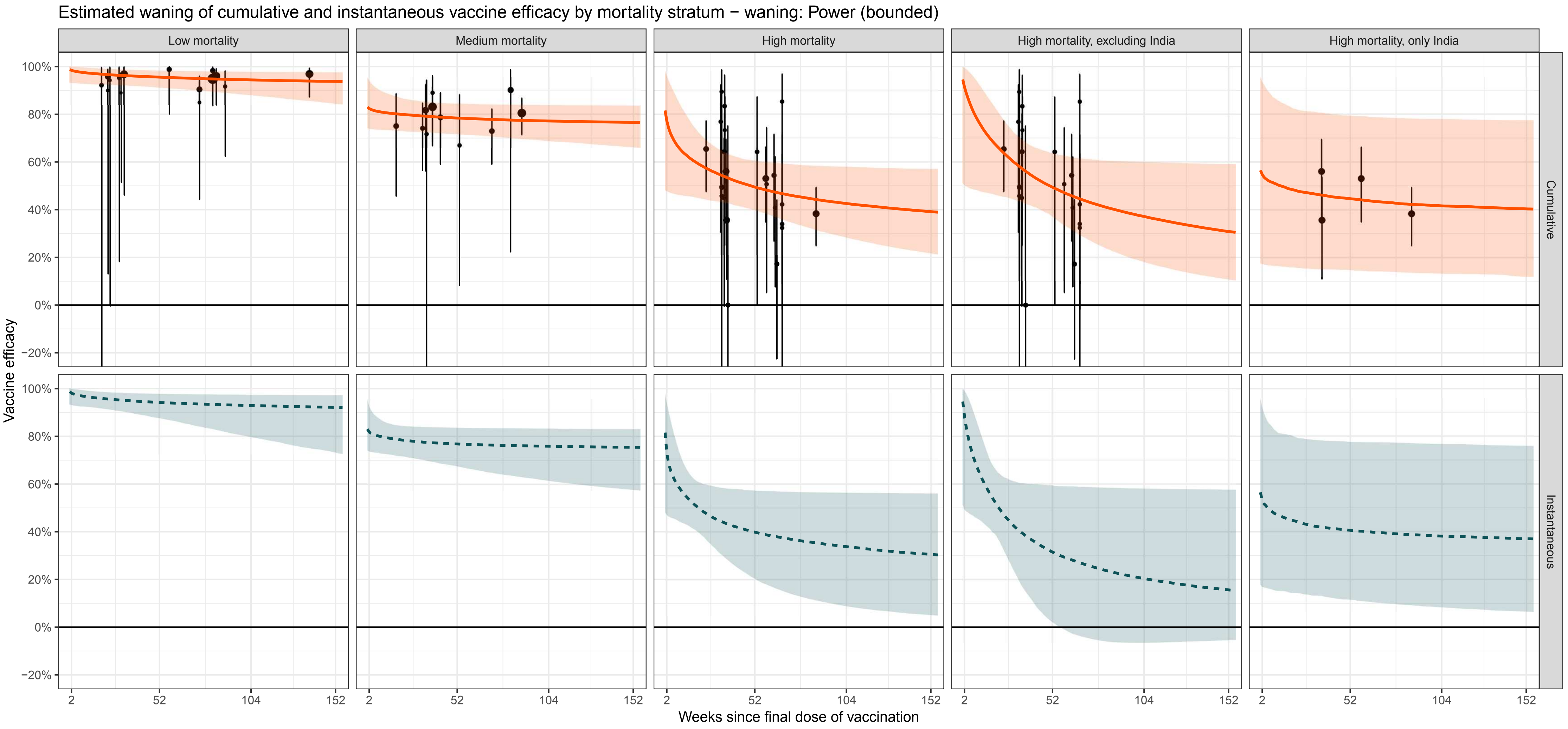

Estimated waning of cumulative and instantaneous vaccine efficacy by mortality stratum − waning: Power (bounded)
Low mortality
Medium mortality
High mortality
High mortality, excluding India
High mortality, only India
Cumulative
Instantaneous
Vaccine efficacy
100%
80%
60%
40%
20%
0%
−20%
2
52
104
152
Weeks since final dose of vaccination

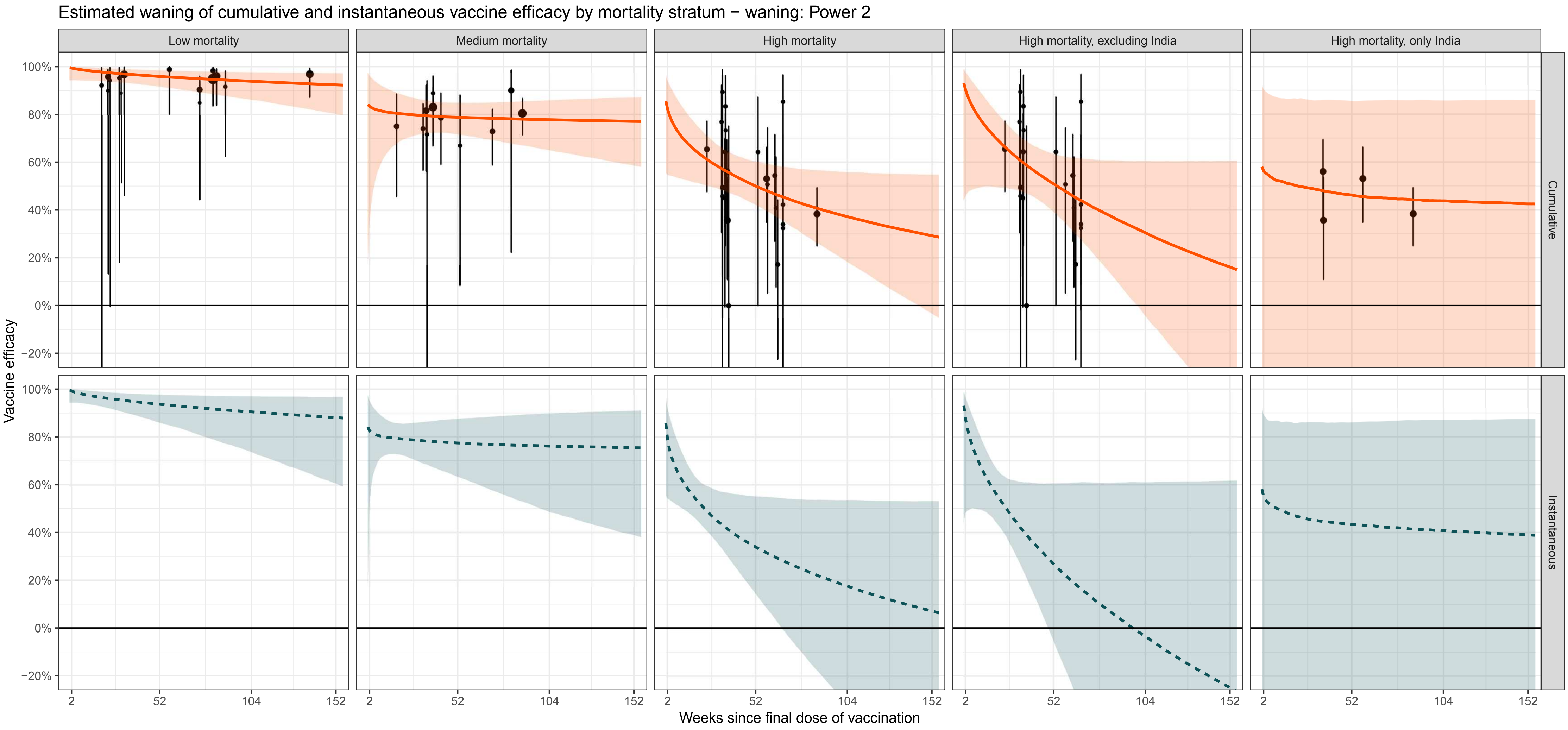
Estimated waning of cumulative and instantaneous vaccine efficacy by mortality stratum − waning: Power 2
Low mortality
Medium mortality
High mortality
High mortality, excluding India
High mortality, only India
Cumulative
Instantaneous
Vaccine efficacy
100%
80%
60%
40%
20%
0%
−20%
2
52
104
152
Weeks since final dose of vaccination

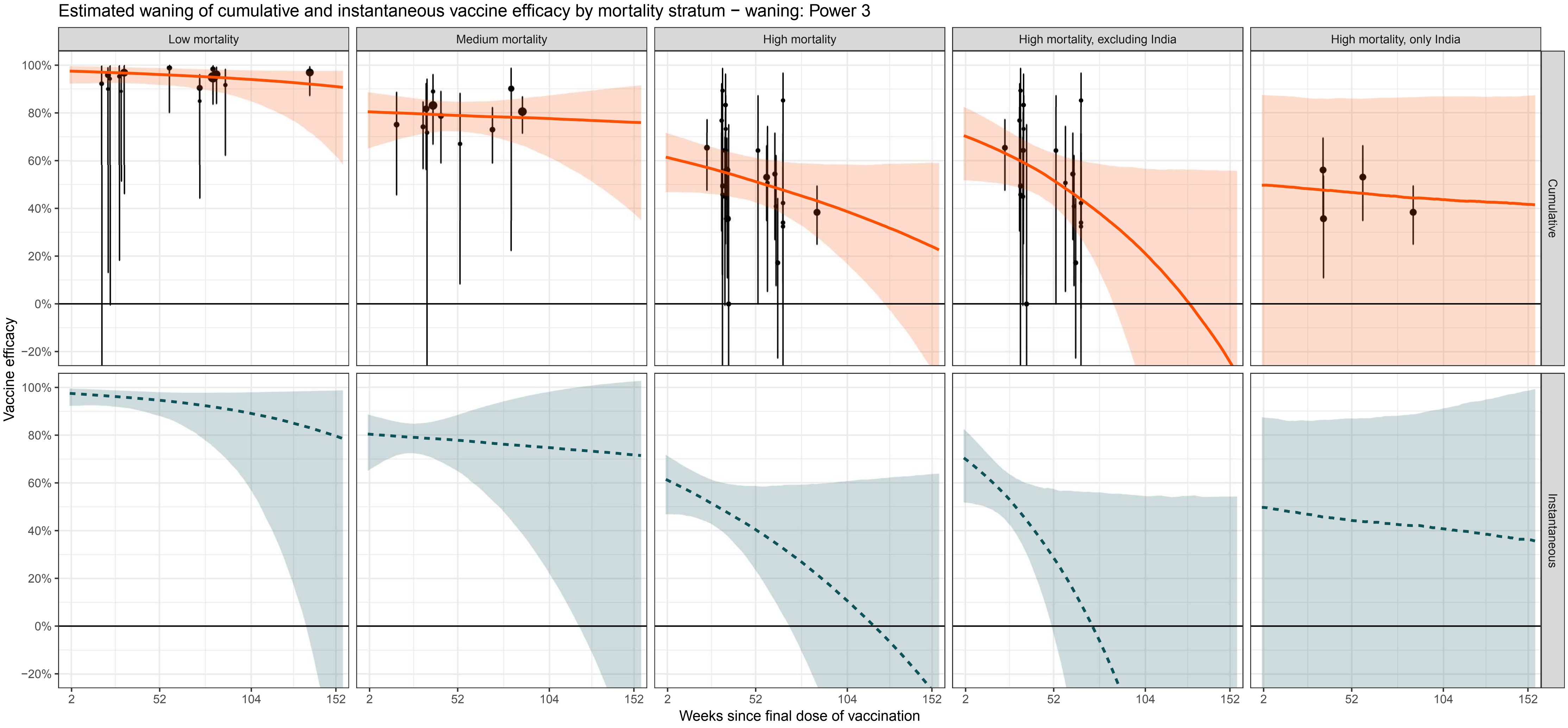

Estimated waning of cumulative and instantaneous vaccine efficacy by mortality stratum − waning: Power 3
Low mortality
Medium mortality
High mortality
High mortality, excluding India
High mortality, only India
Cumulative
Instantaneous
Vaccine efficacy
100%
80%
60%
40%
20%
0%
−20%
2
52
104
152
Weeks since final dose of vaccination

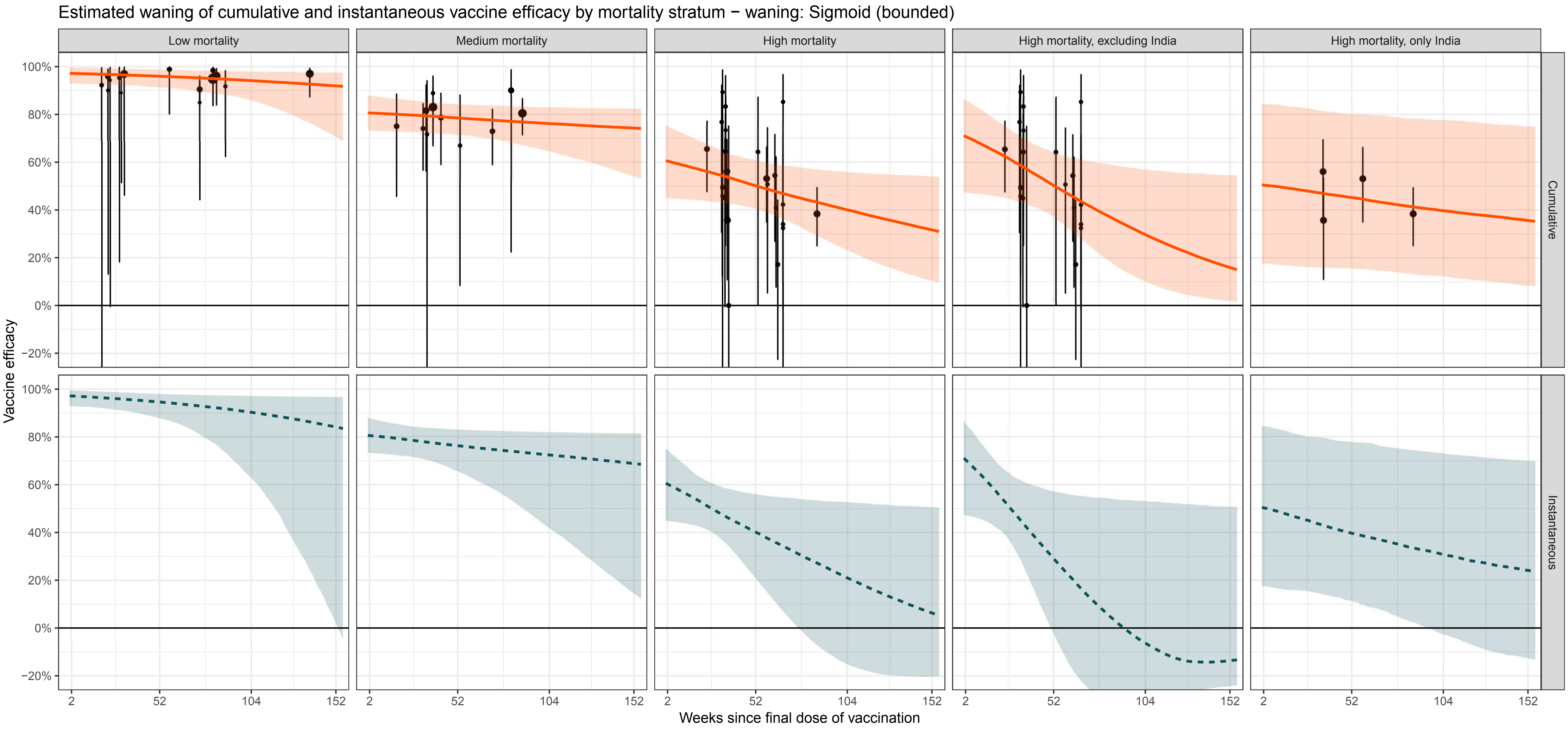

Estimated waning of cumulative and instantaneous vaccine efficacy by mortality stratum − waning: Sigmoid (bounded)
Low mortality
Medium mortality
High mortality
High mortality, excluding India
High mortality, only India
Cumulative
Instantaneous
Vaccine efficacy
100%
80%
60%
40%
20%
0%
−20%
2
52
104
152
Weeks since final dose of vaccination

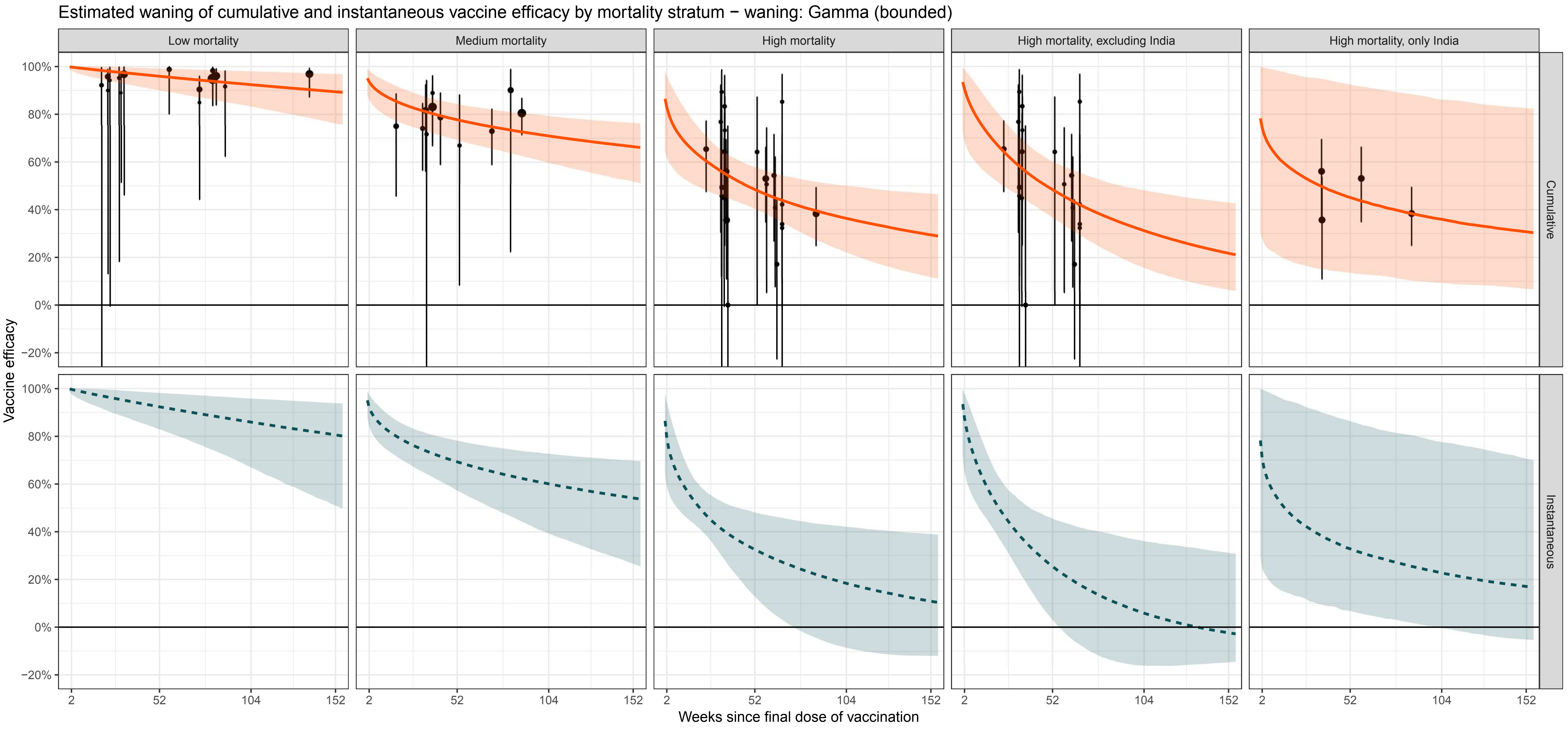

Estimated waning of cumulative and instantaneous vaccine efficacy by mortality stratum − waning: Gamma (bounded)
Low mortality
Medium mortality
High mortality
High mortality, excluding India
High mortality, only India
Cumulative
Instantaneous
Vaccine efficacy
100%
80%
60%
40%
20%
0%
−20%
2
52
104
152
Weeks since final dose of vaccination

3.2.2 Visual comparison of alternative waning functions in head-to-head trial (RV3-BB Indonesia)

The next pages show plots with the fitted VE and their corresponding iVE by weeks since final dose of vaccination for alternative waning functions in the head-to-head trial of RV3-BB in Indonesia. They correspond to Figure 2 in the main paper of this publication.

Each figure shows the median and 95% credible intervals of cumulative and instantaneous vaccine efficacy by duration of follow-up and type of schedule (neonatal vs infant) following three doses of RV3-BB in Indonesia. *Data points shown in the left-hand panels represent observed vaccine efficacies derived from cumulative Kaplan-Meier hazard ratios, and error bars their corresponding 95% confidence intervals. Solid lines and dashed lines represent medians. Shaded areas represent 95% credible intervals.*

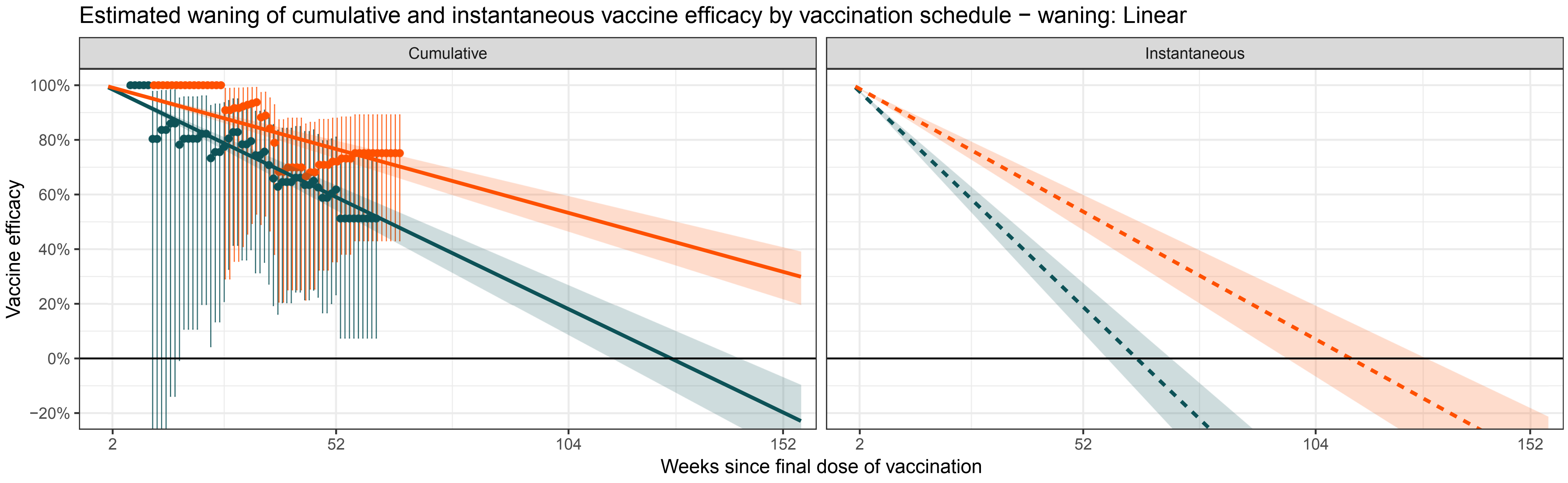
Estimated waning of cumulative and instantaneous vaccine efficacy by vaccination schedule − waning: Linear
Cumulative
Instantaneous
Vaccine efficacy
100%
80%
60%
40%
20%
0%
−20%
2
52
104
152
Weeks since final dose of vaccination

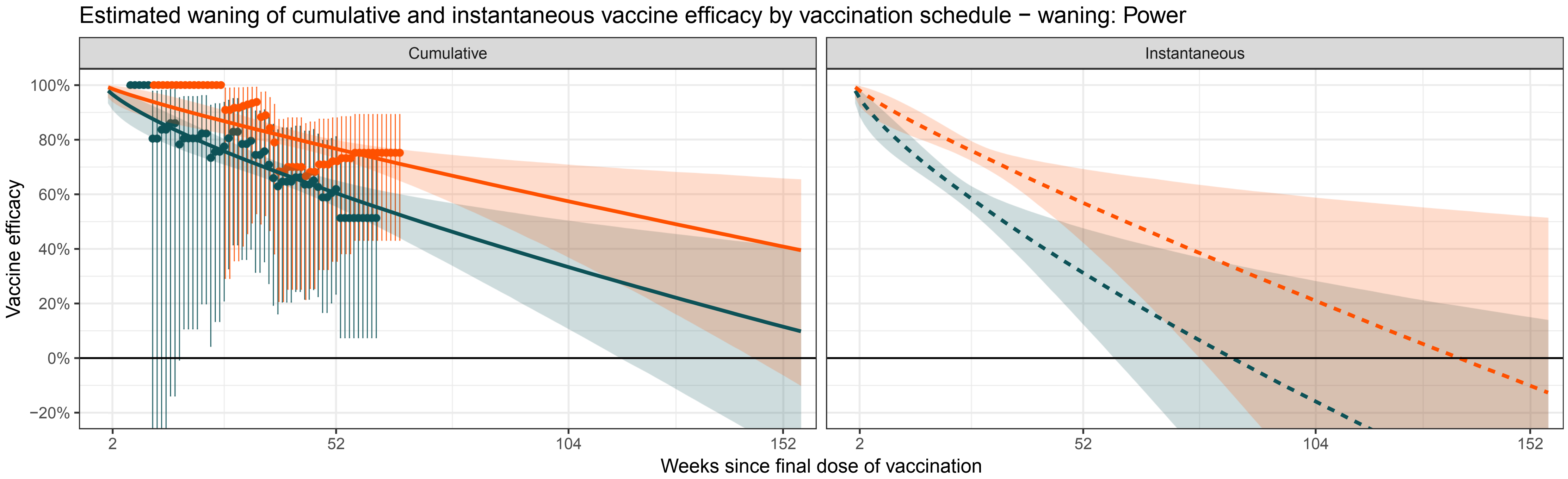

Estimated waning of cumulative and instantaneous vaccine efficacy by vaccination schedule − waning: Power
Cumulative
Instantaneous
100%
80%
60%
40%
20%
0%
−20%
Vaccine efficacy
2
52
104
152
Weeks since final dose of vaccination

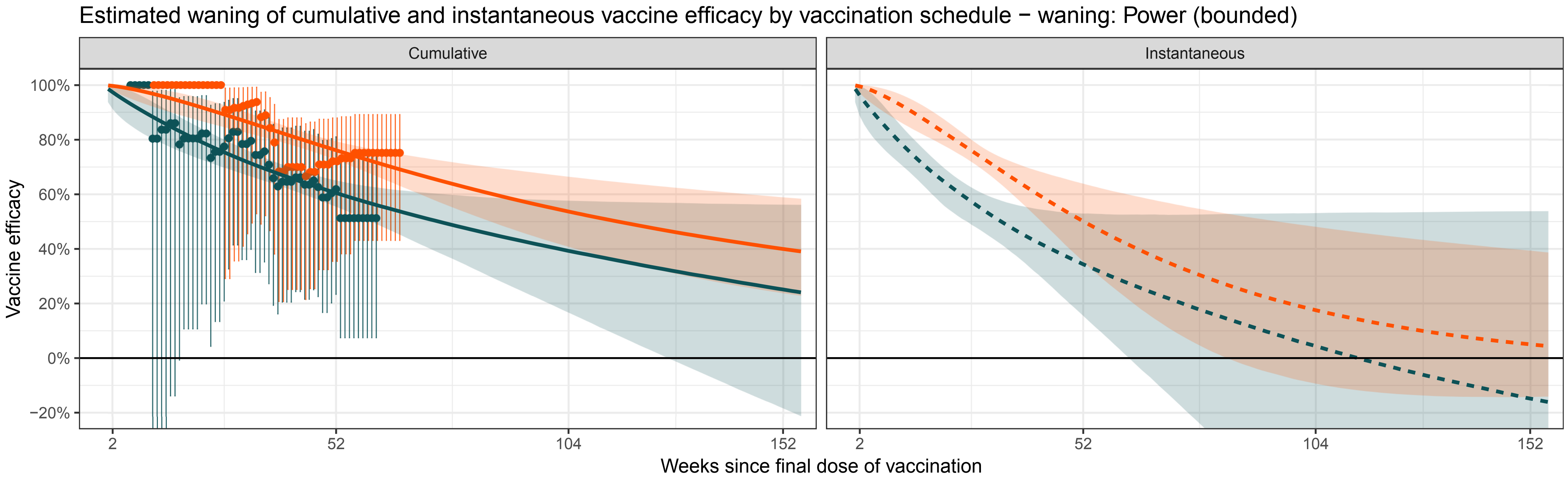

Estimated waning of cumulative and instantaneous vaccine efficacy by vaccination schedule − waning: Power (bounded)
Cumulative
Instantaneous
100%
80%
60%
40%
20%
0%
−20%
Vaccine efficacy
2
52
104
152
Weeks since final dose of vaccination

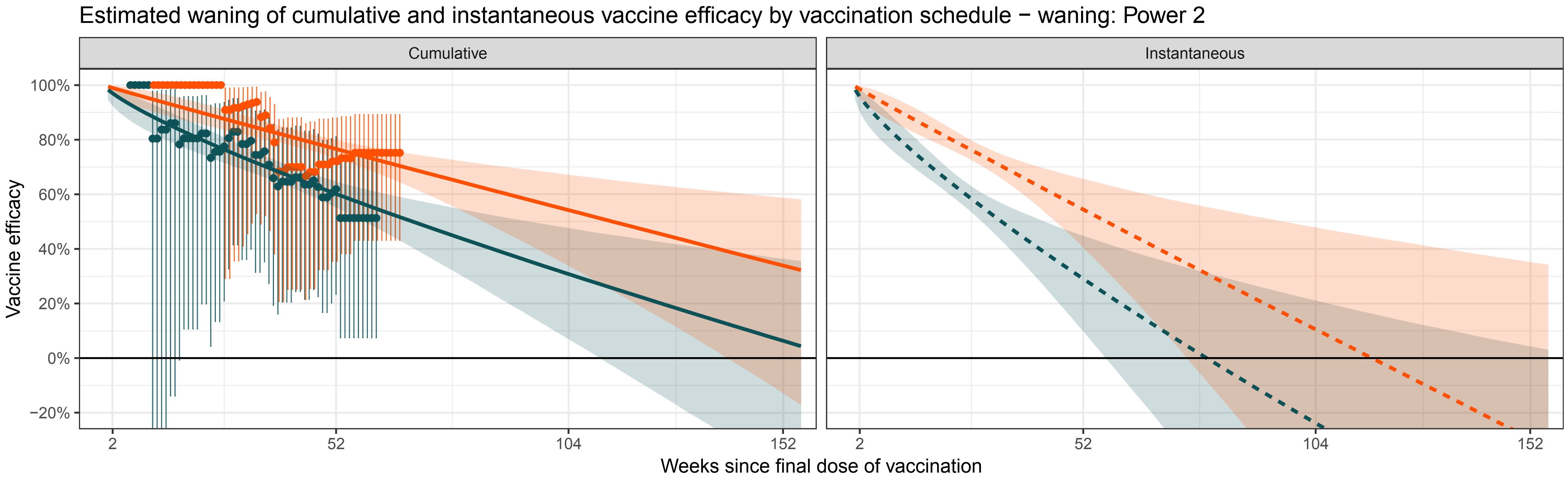

Estimated waning of cumulative and instantaneous vaccine efficacy by vaccination schedule − waning: Power 2
Cumulative
Instantaneous
100%
80%
60%
40%
20%
0%
−20%
Vaccine efficacy
2
52
104
152
Weeks since final dose of vaccination

Estimated waning of cumulative and instantaneous vaccine efficacy by vaccination schedule − waning: Power 3

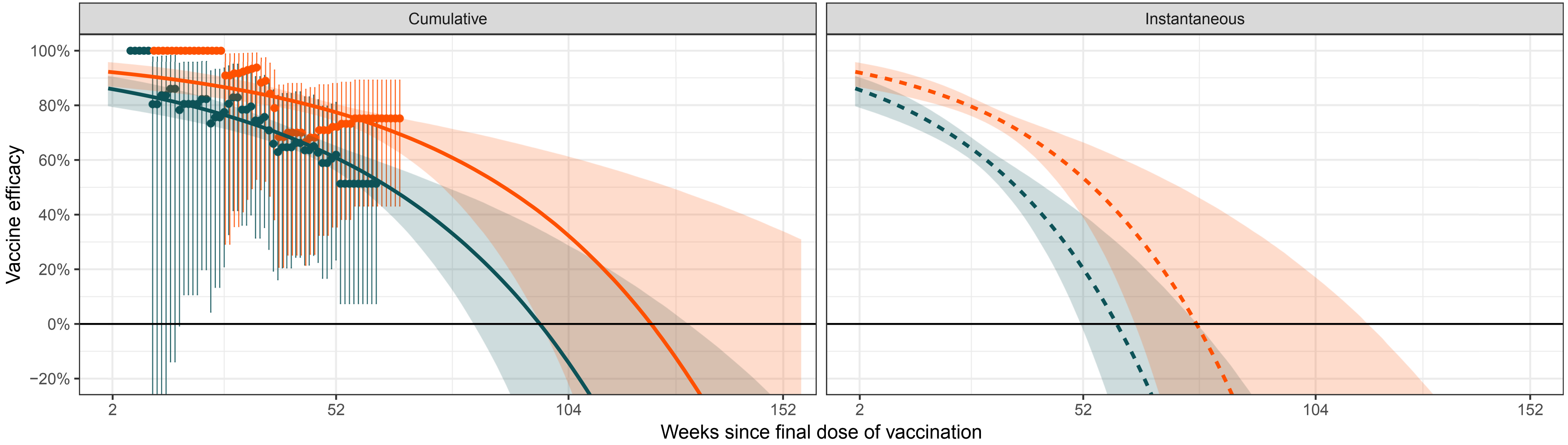

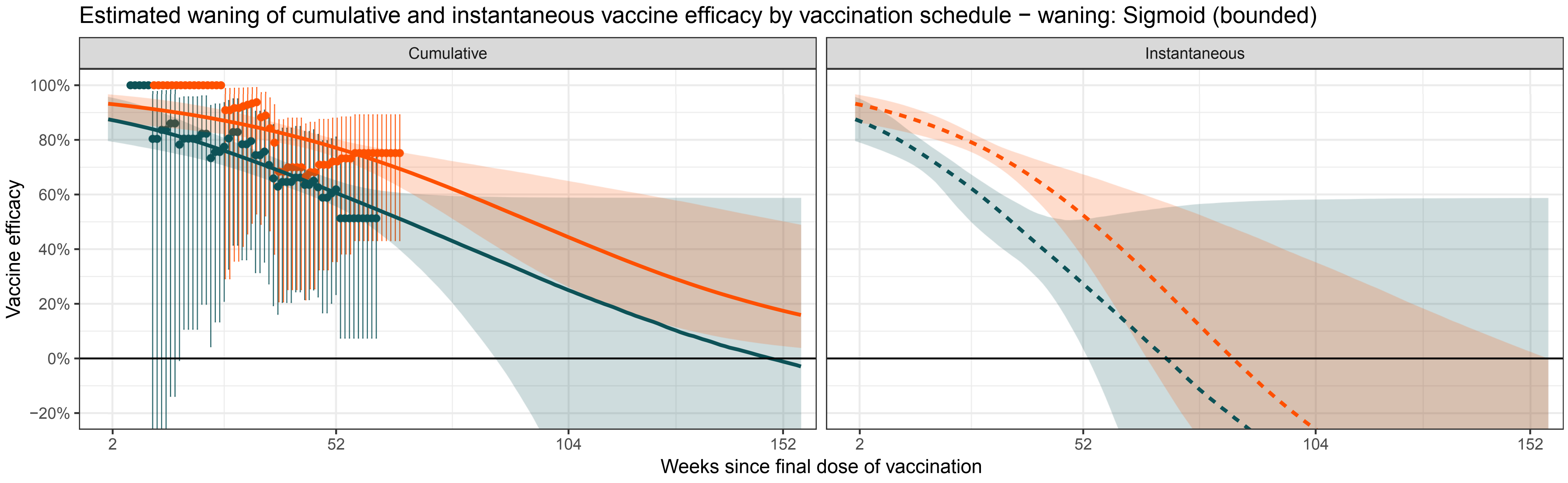

Estimated waning of cumulative and instantaneous vaccine efficacy by vaccination schedule − waning: Sigmoid (bounded)
Cumulative
Instantaneous
Vaccine efficacy
100%
80%
60%
40%
20%
0%
−20%
2
52
104
152
Weeks since final dose of vaccination

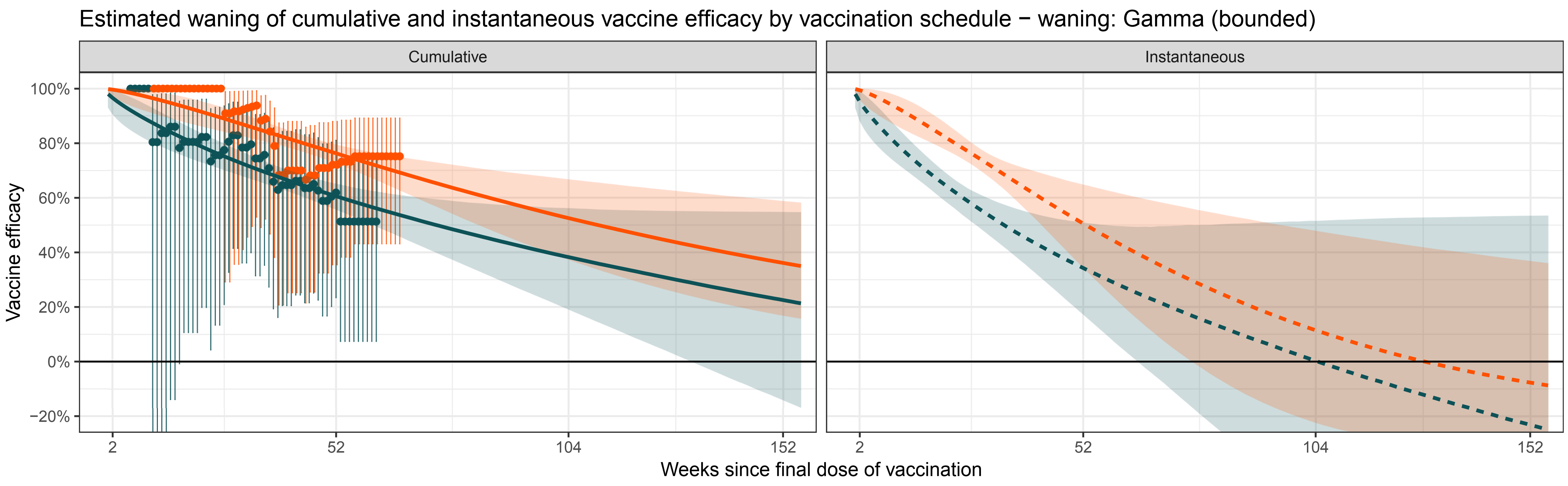

Estimated waning of cumulative and instantaneous vaccine efficacy by vaccination schedule − waning: Gamma (bounded)
Cumulative
Instantaneous
Vaccine efficacy
100%
80%
60%
40%
20%
0%
−20%
2
52
104
152
Weeks since final dose of vaccination

4. Sensitivity analysis for the high-mortality stratum

The plot below shows median and 95% credible intervals of cumulative and instantaneous vaccine efficacy by duration of follow-up after two or three doses of oral rotavirus vaccination (infant schedules only) in high-mortality settings: sensitivity analysis showing results with and without the Indian data points, and for India alone.

As mentioned in our paper, we found no evidence that iVE significantly differed after 2 weeks or 12 months of follow-up when excluding the Indian data points from the high-mortality stratum.

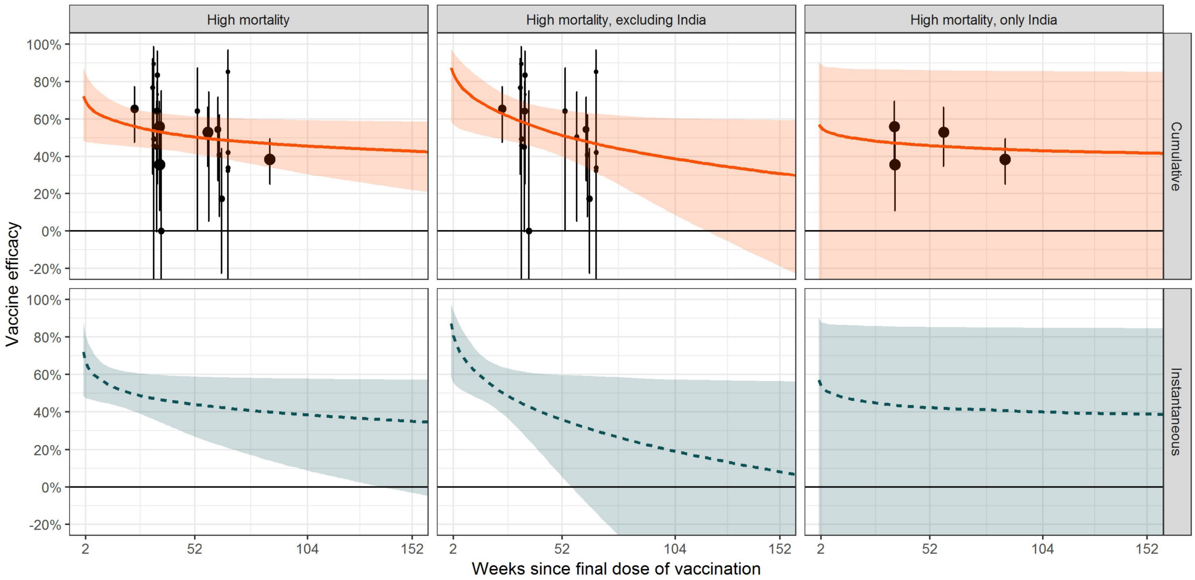

A simple power function was used to represent vaccine waning over time. See Section 3 for equivalent plots based on other potential waning functions.

5. References

[1] Smith PG, Rodrigues LC and Fine PEM. Assessment of the Protective Efficacy of Vaccines against Common Diseases Using Case-Control and Cohort Studies. *International Journal of Epidemiology* 1984; 13(1):87-93

[2] Bines JE, At Thobari J, Satria CD, et al. Human Neonatal Rotavirus Vaccine (RV3-BB) to Target Rotavirus from Birth. *N Engl J Med* 2018;378(8):719-730.
